# Progressive lifespan modifications in the corpus callosum following a single concussion in juvenile male mice monitored by diffusion MRI

**DOI:** 10.1016/j.expneurol.2025.115455

**Published:** 2025-09-07

**Authors:** Andre Obenaus, Brenda P. Noarbe, Jeong Bin Lee, Polina E. Panchenko, Fang Tong, Sean D. Noarbe, Claire Bottini, Yu Chiao Lee, Jerome Badaut

**Affiliations:** a Department of Pediatrics, School of Medicine, University of California Irvine, Irvine, CA, USA; b Basic Science Department, Loma Linda University School of Medicine, Loma Linda, CA, USA; c CNRS UMR 5536 RMSB, University of Bordeaux, Bordeaux, France; d CNRS UMR 7372 CEBC, La Rochelle University, Villiers-en-Bois, France

**Keywords:** Magnetic resonance imaging, Diffusion tensor imaging, Closed head injury, Aging, Astrocytes, Microglia, Inflammation

## Abstract

**Introduction:**

The vulnerability of white matter (WM) in acute and chronic moderate-severe traumatic brain injury (TBI) has been established. In concussion syndromes, including preclinical rodent models, lacking are comprehensive longitudinal studies spanning the mouse lifespan. We previously reported early WM modifications using clinically relevant neuroimaging and histological measures in a model of juvenile concussion at one month post injury (mpi) who then exhibited cognitive deficits at 12mpi. For the first time, we assess corpus callosum (CC) integrity across the lifespan after a single juvenile concussion utilizing diffusion MRI (dMRI).

**Methods:**

C57Bl/6 mice were exposed to sham or two severities of closed-head concussion (Grade 1, G1, speed 2 m/s, depth 1 mm; Grade 2, G2, 3 m/s, 3 mm) using an electromagnetic impactor at postnatal day 17. In vivo diffusion tensor imaging was conducted at 1, 3, 6, 12 and 18mpi and processed for dMRI parametric maps: fractional anisotropy (FA), axial (AxD), radial (RD) and mean diffusivity (MD). Hemispheric CC and regional CC data were extracted. To identify the biological basis of altered dMRI metrics, astrocyte and microglia in the CC were characterized at 1, 12 and 18 mpi by immunohistochemistry.

**Results:**

Hemispheric CC analysis revealed altered FA and RD trajectories following juvenile concussion. Shams exhibited a temporally linear increase in FA with age while G1/G2 mice had plateaued FA values. G2 concussed mice exhibited high variance of dMRI metrics at 18mpi, which was attributed to the heterogeneity of TBI on the anterior CC. Regional analysis of dMRI metrics at the impact site unveiled significant differences between G2 and sham mice. The dMRI findings appear to be driven, in part, by loss of astrocyte morphology.

**Conclusion:**

For the first time, we demonstrate progressive perturbations to WM of male mice after a single juvenile concussion across their lifespan. The CC alterations were dependent on concussion severity with elevated sensitivity in the anterior CC that was related to astrocyte and microglial morphology changes. Our findings suggest that long-term monitoring of children with juvenile concussive episodes using dMRI is warranted, focusing on vulnerable WM tracts.

## 1. Introduction

Concussion awareness has increased considerably over the last decade particularly in sports-related brain injuries (Snyder and Giza, 2019). Altered brain connectivity is a signature of concussion injuries both in the acute and in the chronic epochs (Hayes et al., 2016; Onicas et al., 2023; Bouchard et al., 2023). Broadly, these studies report decrements in structural (diffusion magnetic resonance imaging (MRI)) and functional (evoked or resting state MRI) connectivity between affective, motor and somatic brain regions. Pan and colleagues demonstrated that structural and functional connectivity (coupling) is disrupted in sensorimotor and cognitive circuits after mild traumatic brain injury (mTBI) (Pan et al., 2023). In pediatric mTBI there is sensitivity to WM tracts, with increased mean diffusivity (MD) in anterior thalamic radiations, arcuate fasciculus and superior longitudinal fasciculus that was dependent on time post injury (Ware et al., 2022). The corpus callosum (CC) has significant cortical and interhemispheric projections and in mTBI patients progressive alterations were reported in the CC from the subacute to the late chronic (12 month post injury; mpi) time point (Wang et al., 2021). Moreover, in TBI patients WM loss co-exists with neuroinflammation after a single injury episode (Wu et al., 2020). Thus, WM in pediatric and adult mTBI patients is particularly vulnerable to loss of integrity and appears to undergo progressive long-term alterations.

In rodent models, WM injury has been assessed in several TBI models and at various time points. No behavioral changes have been described within the 14dpi experimental period post-mTBI with a range of severities; however, increased neurodegeneration in the CC has been noted (Velayudhan et al., 2022; Marion et al., 2018). In a mild fluid percussion model (FPI) of brain injury, mice did not exhibit decreased numbers of oligodendrocytes nor their progenitors in the CC at acute 1 or 3dpi time points (Adams et al., 2023). The authors noted that the number of mature oligodendrocytes were reduced at 3dpi with apparent sparing of myelin. Nodal proteins flanking nodes of Ranvier were also decreased in the CC, consistent with presumed altered function (Adams et al., 2023) and exhibited progressive temporal deficits leading to altered conduction (Marion et al., 2018). Similarly, in a mild repeated cortical contusion injury (CCI) model we reported altered myelin structure at 60dpi with fragmented myelin sheaths (Donovan et al., 2014). Thus, there are clear alterations to the CC in various models of mild TBI at acute and chronic time points that are likely to impact connectivity.

Clinically, as noted above, non-invasive MRI can track altered WM composition and connectivity. Diffusion MRI (dMRI) acquired with multiple tensors and with multiple b values can be utilized to assess WM integrity in rodent models of TBI (as reviewed in (Hutchinson et al., 2018)). A temporal study utilizing the weight drop model in adult mice demonstrated transient dMRI alterations in the CC from approximately 7–21dpi that then resolved by 38dpi (Qin et al., 2018). In adult mice exposed to a mild CCI we reported that by 60dpi axial (AxD) and radial (RD) diffusivity decreased in the CC after a single mTBI, but elevations in these dMRI metrics were observed after repeated mTBI (Donovan et al., 2014). At even longer time points, Moro and colleagues reported chronic reductions in CC volumes, decreased FA and AxD, with increased RD at both 6 and 12mpi in a repeated concussion model (Moro et al., 2023). These altered dMRI metrics were coincident with decreased performance in cognition tests at 12mpi, like those we previously reported after a single concussion (Rodriguez-Grande et al., 2018). Thus, there is clear evidence morphologically and from dMRI studies that early injury to the CC results in long-term changes in WM microstructure.

However, lacking in the literature are studies that monitor the WM in the brain longitudinally after a single concussion in a juvenile rodent model that mimics the human condition. We have in part addressed these gaps with some of our publications as summarized in Table 1. The induction of injury of the mouse at postnatal day 17 (PND17) was selected based on its approximate correspondence to 8–10 yr old humans and corresponds with the peak of human myelination (Semple et al., 2013). We recently developed a model that includes a lateral rotational aspect after a single impact to one hemisphere that was previously well characterized (Rodriguez-Grande et al., 2018). Early MRI changes were noted in the CC up to 1mpi and cognitive alterations were observed at 12mpi (Obenaus et al., 2023). We hypothesized that WM, in particular, the CC would exhibit progressive alterations in dMRI metrics related to chronic inflammation. To assess the temporal evolution of the dMRI metrics over the lifespan of the mouse, we undertook dMRI at 7 distinct epochs up to 18mpi along with histological assessments of astrocytes and microglia, at 1, 12 and 18mpi. Here, we report progressive alterations to the CC after a single concussion to the juvenile mouse (PND17 injury). These studies provide the basis for future studies investigating therapeutic interventions to ameliorate the advancing WM decrements in TBI patients.

## 2. Materials and methods

### 2.1. Animals

C57BL/6 J were bred in–house with breeder mice purchased from Janvier (Le Genest-Saint-Isle, France). Animals were maintained at 21 °C ± 1 °C, 55 % ± 10 % humidity, in a 12-h light-dark cycle with access to food and water ad libitum. Mild traumatic brain injury (mTBI) was induced in male pups at PND17. Males were utilized given the higher incidence of TBI in male children (Coronado et al., 2011). Animals were randomly assigned (using weight-matching) to sham or one of the two mTBI groups with differing severities (Grade 1, G1 or Grade 2, G2) with a minimum sample size of 8 mice per group for a total of *n* = 27–35 per time point (Supplementary Table 1 A). Mice were weaned at PND 25 and housed in groups of 3 to 5 individuals. All animal procedures were carried out following the European Council directives (86/609/EEC), the Animal Research: Reporting of in vivo Experiments (ARRIVE) guidelines and local ethics Committee (APAFIS#19296–201,902,191, 637,994.v4).

### 2.2. Closed-head injury

**Fig. 1.**
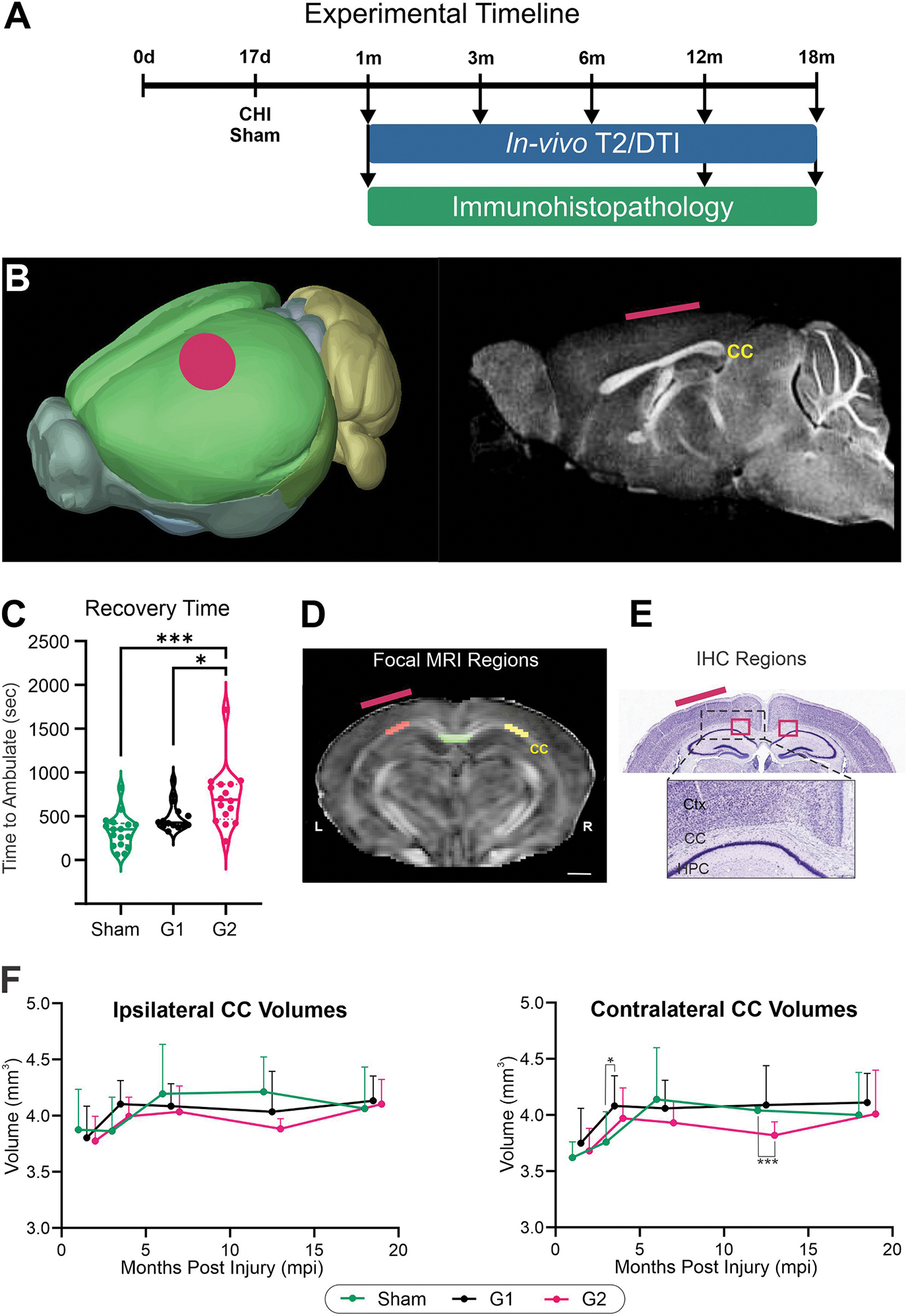
Experimental timeline and regions of interest. A) Experimental timeline using a closed head injury (CHI) at post-natal day 17 illustrates the neuroimaging time points (from 1 to 18 months post injury, mpi) with correlative immunohistopathology at 1, 12 and 18mpi. B) Schematic brain showing the location of the CHI (red dot) (left panel) and a sagittal MRI image demarcating the corpus callosum (CC) location and the location of the CHI (red line) (right panel). C) Recovery of ambulation immediately after CHI showed significant increased times in G2 compared to G1 mice and shams (one-way ANOVA with post-hoc *t*-tests, *p < 0.05, ***p < 0.0001 compared to Sham). D) Manually drawn MRI regions of interest at the level of the CHI with ipsilateral (yellow) and contralateral (red) CC regions for focal analysis. The center CC where the two hemispheres join was also analyzed (green). (see Supplementary Fig. 1 for automated AMBC atlas-derived CC regions). E) Nissl-stained section illustrating the CC regions situated between Ctx and hippocampus (HPC) analyzed from immunohistochemical sections. (red line indicates location of CHI). F) Ipsilateral and contralateral CC volumes plotted for each month post injury (mpi) for Sham, G1 and G2 mice cohorts. (Mixed effects modeling with Tukey’s multiple comparisons, *n* = 9–12/group/time point, *p < 0.05, ***p < 0.0001 compared to Sham,).

A mild closed-head injury (CHI) with 2-severity grades (G1, G2) model was utilized as previously described (Rodriguez-Grande et al., 2018; Obenaus et al., 2023; Clément et al., 2020). This clinically relevant model encompasses rotational head movement, requires no surgery to expose the cranium, and incorporates two injury severities to titrate concussive events, better reflecting the inherently heterogeneous nature of mTBI. CHI was administered at PND 17 (Fig. 1A, B). In short, mice were anesthetized using 2.5 % isoflurane and 1.5 l/min air for exactly 5 min and then placed on a sheet of tin foil under the impactor tip (3 mm round tip). Without surgery, animals received a focal impact directly over the left somatosensory-parietal cortex center (Bregma approximately −1.7 mm anterior-posterior (AP) coordinate; medial-lateral (ML) coordinate 1.5 mm) using an electromagnetic Leica Impact One stereotaxic impactor (Leica Biosystems, Richmond, IL, USA) with following parameters: G1 = speed = 2 m/s, depth = 1 mm, dwell time = 100 ms; G2 = speed = 3 m/s, depth = 3 mm, dwell time = 100 ms (Fig. 1B). The hair on the scalp was not removed and there were no instances of mortality or skull fractures at either severity which were confirmed on MRI and at time of perfusion fixation. Sham animals underwent the same anesthesia procedure and were placed under the impactor apparatus but did not receive an impact. Each litter was divided into sham, G1 and G2 groups and after impact mice were allowed to fully recover in individual cages until reappearance of exploratory behaviors and were returned to their original home cage with their respective dam and litter. Times to recover ambulation immediately after injury were significantly increased in G2 mice compared to G1 and sham mice (one way ANOVA, (*p < 0.05, ***p < 0.0001 compared to Sham) (Fig. 1C). We have previously reported on the consistency of induction in this model across experimental sites (Dubois et al., 2025). Our previous study demonstrated that the G1 depth (1 mm) never exhibited MRI observable alterations whereas about 20–30 % of mice exhibited extravascular blood with the more severe G2 depth (3 mm) (Rodriguez-Grande et al., 2018). As noted, no skull fractures or other adverse events were observed at either depth. The two severities in this study were utilized to examine the long-term effects of a milder versus more severe concussion.

### 2.3. MR imaging

Our longitudinal design included 1 (actual age = 47d), 3-, 6-, 12-, and 18-months post impact (mpi) time points, involving the same cohort of animals examined in vivo throughout all the time points for consistent and comparable outcome measurements (Fig. 1A). In vivo longitudinal DTI data were acquired on a 7 T scanner (Bruker Biospin, Billerica, MA) with the following parameters: repetition time (TR)/Echo time (TE) = 1000 ms/30 ms, 0.267 mm slice thickness, 21 diffusion gradient directions, 1.6 cm × 1.28 cm field of view (FOV), 164 × 128 acquisition matrix, b = 2000 mT/m, and 2 b0 images acquired prior to weighted images. T2-weighted imaging (T2WI) was also acquired to extract regional and brain volumes using the following parameters: TR/TE = 3000 ms/7 ms, 1.6 cm × 1.28 cm FOV, 162 × 128 acquisition matrix, 0.8 mm slice thickness, and 25 echoes. All scans were acquired using a Bruker mouse surface coil and total imaging time was 1 h 45 min.

### 2.4. MR analysis

Acquired MR images underwent preprocessing to optimize image quality and minimize artifacts as previously described (Wendel et al., 2018). The first echo of T2WI and the mean image of DTI b0s were utilized for brain extraction and registration. The skull was stripped from the brain in a semi-automated manner initially via masks generated using 3D Pulse-Coupled Neural Networks (PCNN3D v1.2) which were then reviewed and adjusted by a blinded experimenter (Chou et al., 2011). Once the brain was isolated, T2WI data underwent N4 bias field correction with Advanced Normalization Tools (ANTs v2.1). Diffusion data underwent eddy current correction and DTI metrics were reconstructed using FMRIB Software Library’s DTIFIT. DTI parametric maps of fractional anisotropy (FA), axial diffusivity (AxD), mean diffusivity (MD), radial diffusivity (RD) and eigenvalues (L1,L2,L3) were obtained.

3D automatic registration was used for brain structure delineation (Clément et al., 2020). FMRIB’s Linear Image Registration Tool (FLIRT) was utilized to register the Australian Mouse Brain Mapping Consortium (AMBMC) model-based atlas to the T2/DTI native space (Jenkinson et al., 2002). The output transformation matrix was then applied to our atlas using ANTs Symmetric Normalization (SyN) algorithm (Jenkinson et al., 2002). This robust method not only utilizes many of the same tools and methodologies utilized in clinical MR data processing but allows for unbiased and rapid segmentation of the corpus callosum and other regions of interest. DTI and volumetric (T2WI) data were extracted using the transformed atlas labels (FSL v5.0; FMRIB, Oxford, UK) (Andersson and Sotiropoulos, 2016; Woolrich et al., 2009). Exemplar whole hemispheric CC regions of interest on T2 images are shown in Supplementary Fig. 1.

An additional level of analysis utilized manual regions of interest drawn on the corpus callosum on 2 contiguous slices centered at the impact site (Fig. 1D). Delineations were drawn using DSI studio (April 11, 2018 build; http://dsi-studio.labsolver.org). DTI metrics (AxD, MD, RD, FA) were extracted from the regions of interest and summarized in MS Excel.

### 2.5. Immunohistochemistry (IHC)

Sham or G2 severity mTBI mice were examined at 1 (*n* = 3–4 per group); 12 (n = 3–4 per group) and 18 mpi (*n* = 7–8 per group) for histology. After a transcardiac perfusion with 4 % paraformaldehyde prepared in a PBS, brain tissue was collected. Brain tissue was collected from random mice in the cohort and processed as published previously (Obenaus et al., 2023; Clément et al., 2020). Sections of 50 μm thickness were cut using the vibratome (Leica, Richmond, IL, USA) in the coronal plane and stored at −20 °C for long-term storage in a cryoprotective medium (30 % ethylene glycol and 20 % glycerol in PBS). Two sections per animal (between Bregma −1.3 mm and − 2 mm) were used for free-floating IHC. Antigen retrieval was performed to restore the binding of primary antibody to the epitope of interest with sections incubated in a mixture of 1/3 acetic acid and 2/3 of absolute ethanol for 10 min at −20 °C and then extensively washed in PBS (6 × 15 min). To saturate nonspecific antigen-binding sites, the sections were then incubated in blocking solution composed of 1 % BSA and 0.3 % Triton X-100 in PBS for 2 h at room temperature (RT). Brain sections were incubated overnight at 4 °C with the following primary antibodies diluted in blocking solution: chicken polyclonal anti-mouse glial fibrillary acidic protein (GFAP, Abcam ref. ab4674, RRID AB_304558, 1:3000), rabbit polyclonal anti-mouse Ionized calcium-binding adaptor molecule 1 (IBA1, Wako ref. 019–19,741, RRID AB_839504, 1:1500) and rabbit polyclonal anti-mouse neurofilament-200 (NF200, Sigma-Aldrich ref. N4142, RRID AB_477272, 1:500). The following day slices were washed with PBS (2 × 10 min) and incubated for 1.5 h at RT with the corresponding secondary fluorescent antibodies diluted 1:1000 in blocking solution: goat anti-chicken Alexa Fluor^™^ 568 nm (Invitrogen ref. A-11041, RRID AB_2534098), goat anti-rabbit Alexa Fluor^™^ 647 nm (Invitrogen ref. A-21244, RRID AB_2535812), goat anti-rabbit Alexa Fluor^™^ 488 nm (Invitrogen ref. A-11034, RRID AB_2576217). Slices were washed in PBS (3 × 10 min), mounted on slides and cover-slipped using Vectashield antifade mounting medium with 4′,6-diamidino-2-phenylindole (DAPI, Vector laboratories ref. H-1200, Newark, CA, USA) and stored at 4 °C.

### 2.6. IHC image acquisition

We acquired 3 fields of view (FOV) from the CC with 3 regions of interest and from two sections resulting in a total of 18 measurements/mouse. The values were then averaged together for each mouse. Images were acquired using Nikon Eclipse 90i epifluorescence microscope with attached DS-Qi1Mc camera (Nikon Europe, Amstelveen, The Netherlands), pE-300^white^ CoolLED light source (CoolLED, Andover, UK) and NIS-Elements imaging software (Nikon, version 4.30.02). Coronal brain sections were imaged with 4× and 20× objectives at the level of the lateral CC (Fig. 1E). All acquisition settings of the epifluorescence microscope and software were kept identical within each set of experiments. A negative control staining experiment without the primary antibody showed no detectable signal. Additionally, one z-stack per animal was taken with 40× objective for further fractal analysis. Individuals undertaking the acquisition and analyses were blinded to condition.

### 2.7. Immunohistochemistry analysis

Image analysis was performed using ImageJ software (https://imagej.net/ij/download.html, version 1.54f) (Schindelin et al., 2012). Both experimenters (CB, PP) were blinded to the experimental groups. Immunolabeling intensity was quantified on raw immunofluorescent images taken with 20× objective from ipsilateral and contralateral corpus callosum at the level of the impact site (Supplementary Fig. 2). The areas inside of the corpus callosum were delineated using the polygon tool using the three channels (GFAP, IBA1 and DAPI) and then subsequently removed from all channels. Mean gray values of the CC ROI were measured at a single focal plane.

### 2.8. Morphometric analyses

Skeleton analysis was performed on 20× stack acquisitions from GFAP- and IBA1- immunolabeled sections as published (Clément et al., 2020; Young and Morrison, 2018). Briefly, z-stack images were projected in 2D using the z-projection function with maximum intensity, and each channel was then extracted. The background noise was removed using Fast Fourier Transform (FFT) bandpass filter (default settings: filter small structures up to 3 pixels, large structures down to 40 pixels, no stripe suppression), and unsharp mask filter (radius of 1 pixel and mask weight 0.9). Resulting images were converted to binary using manual threshold function (white cells on black background, using the following threshold 5–7 % for GFAP and 4–5 % for IBA1) with no manual editing. Binary images were skeletonized and then analyzed with ImageJ Analyze Skeleton (2D/3D) plugin (Clément et al., 2020; Arganda-Carreras et al., 2010) without elimination of endpoints (no prune cycle method). The output list of labelled skeletons was reduced by removing cells with structures ≤6 endpoints or ≤4 junctions or structures with ≥100 branches, and skeletons that were not complete cells or skeletons due to artifacts. The morphological features analyzed were number of branches, junctions, and number of slab voxels (converted from pixels to total process length expressed in μm). For statistical analysis, cells were pooled together for each mouse within each experimental group (sham or G2).

Morphological description of positive IBA1-microglia was performed with Fractal analysis to assess microglia (IBA1+) in ipsilateral CC at 12 and 18mpi. We used Fraclac plugin for ImageJ (version 2015Sep090313a9330) using previously published protocols (Young and Morrison, 2018; Karperien et al., 2013; Morrison et al., 2017; Fernandez-Arjona et al., 2019). *Z*-stacks were acquired with 40× objective for increased process details of microglial cells in ipsilateral (G2) or left CC (sham). Four z-projection images from the ipsilateral CC containing IBA1 positive-cells with visible somata were isolated using the polygon tool and duplicated for processing. Individual IBA1-cell pictures were converted to 16-bit, the background was subtracted using a rolling of 100, and contrast was enhanced with a saturation of 0.5 before an unsharp mask was applied with a radius of 2 and a mask of 0.7. Application of a threshold close to 5 % was performed using a despeckle step to remove noise, and conversion of the image into a binary mask followed by a second despeckle step. A manual editing was then performed to connect white dots from the cell using the paintbrush tool, and resultant image was compared to the original. Finally, to remove background particles, the cell was selected with the wand tool and reversion was used to select and erase the background.

A batch mode was used in Fraclac plugin (default settings in box counting: binary, no filters, white background locked, 12 grids in grid design, scaling method with default sampling sizes) to analyze all individual cellular binary images simultaneously. The convex hull (straight line segments joining the outermost foreground pixels) and bounding circle (the smallest circle around the convex hull) were generated for each cell image by the Fraclac plugin. Fractal analysis allows quantification of multiple morphological features of each cell, such as complexity (fractal dimension, D_B_), convex hull span ratio (ratio of major and minor axes of the convex hull; range 0–1), cell area (total number of pixels of the cell converted to μm^2^; one pixel area = 0.0276 μm^2^), cell perimeter (expressed in μm; one pixel side = 0.1661 μm), cell circularity ((4π*cell area) / (cell perimeter)^2^), density (foreground pixels / total number of pixels in convex hull; range 0–1) and others (Morrison et al., 2017; Fernandez-Arjona et al., 2019). Box counting method used by Fraclac determines the amount of pixel detail with increasing scale (Karperien et al., 2013). A higher fractal dimension (D_B_ ranges from 1 to 2) represents a greater complexity of the microglial cell.

### 2.9. Statistical analysis

MRI data derived regions over/under the first/third quartile 1.5xIQR (interquartile range) were excluded using Microsoft Excel (Wendel et al., 2018). GraphPad Prism 7.00 (for Windows, GraphPad Software, San Diego, California USA, www.graphpad.com) was used for data analysis. For comparisons of single features between two groups, a *t*-test with Welch’s correction was used. For comparisons of a single feature between three groups, one-way ANOVA with Tukey’s post-hoc t-test was utilized. For data with multiple time points or with multiple groups and multiple features, a repeated measures two-way ANOVA with Tukey’s post-hoc t-test was used. A mixed effects model with a Tukey’s multiple comparison testing was utilized in the event of missing data. To analyze the evolution of dMRI metrics and to highlight temporal differences we fit the means from each mouse at each time and then used a non-linear Gompertz growth curve using least squares fit. The curves were modeled using GraphPad Prism 10.00. Principal component analysis (PCA) analysis was used to study the similarities between individuals derived from twelve variables obtained from MRI and glia-immunolabeling quantification. Treatments were included as a categorical supplementary variable. PCA computation and representation were conducted on the GraphPad Prism 10.00. Prior to the analysis, all variables were standardized. PC scores were compared together using non-parametric Mann-Whitney test. Pearson’s correlation testing and linear regressions were used to assess correlations between two parameters. The alpha level was set to 0.05. All data are presented as the mean ± standard error of the mean (SEM) unless otherwise indicated.

## 3. Results

The corpus callosum (CC) is the largest WM structure in the mouse. In the present study we used diffusion MRI (dMRI) to monitor longitudinally across the lifespan male mice after a single concussive event at postnatal day 17 (PND17). We examined in the following sequence, 1) brain and regional volumes from T2-weighted imaging (T2WI), 2) dMRI metrics from the CC (axial, radial, mean diffusivity and fractional anisotropy), 3) quantitative assessment of putative chronic inflammation by glial immunochemistry (IHC) of astrocytes and microglia within the CC, and finally, 4) examination of how a single concussion modifies glial morphology and their influence on dMRI metrics across the lifespan using principal component analyses.

### 3.1. Brain and CC volumes across the lifespan

Whole brain volumes across the mouse lifespan were not significantly different between shams, Grade 1 (G1) and Grade 2 (G2) mice across time when accounting for age and experimental group (*p* = 0.652, two-way ANOVA, Age X Group) (Supplementary Table 1B and accompanying graphical representation). In brain volume comparisons within each group, there were significant effects of time within the G2 group with 1 month post injury (mpi) compared to 3, 6, 12 and 18mpi (*p* < 0.0001, two-way ANOVA) and in sham and G1 groups only 1 vs. 18 mpi had significantly different brain volumes. When ipsi- and contralateral hemisphere volumes were assessed, no significant Age X Group interactions were found. Thus, there were no significant differences in brain volumes between sham and concussion groups spanning their lifespan.

Hemispheric CC volumes exhibited ipsi- and contralateral differences between groups and are summarized in Fig. 1F. The contralateral CC volume showed no significant differences between groups except in G2 mice whose CC volumes were significantly reduced relative to shams at 12mpi. The ipsilateral CC volumes significantly increased by 6.17 % in G1 mice at 3mpi (*p* < 0.05) but reduced in G2 mice at 12mo by 7.69 % (*p* < 0.001) compared to shams (Fig. 1F).

### 3.2. dMRI microstructure: fractional anisotropy (FA)

**Fig. 2.**
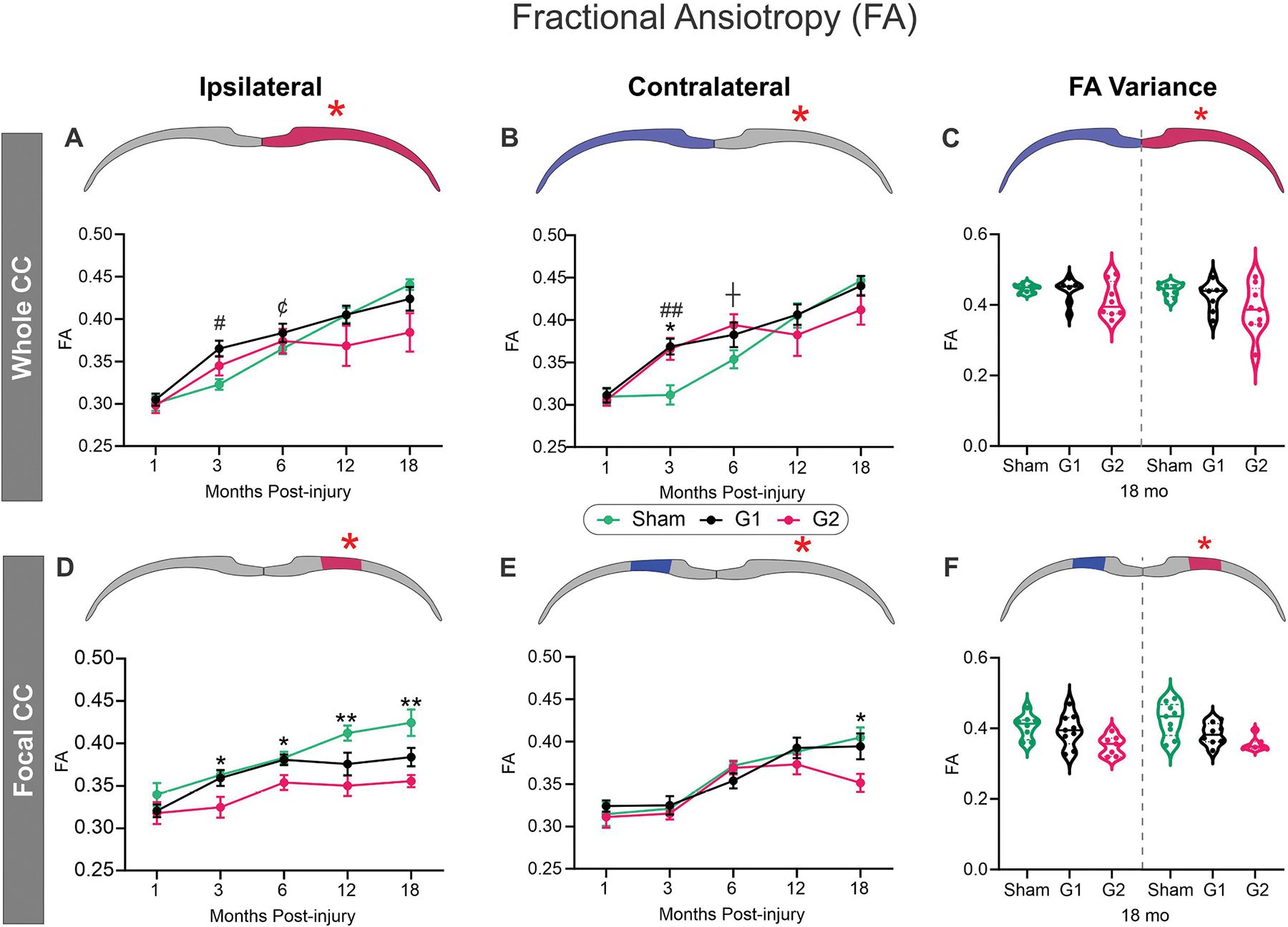
Fractional anisotropy (FA) in the corpus callosum (CC) is reduced late in life after juvenile closed head injury (CHI) at the impact site (red asterisk). A) The ipsilateral whole hemispheric CC FA was elevated at 3mpi in G1 mice, which continues at 6mpi. B) In the whole contralateral CC, both G1 and G2 mice showed early elevated FA at 3mpi. In G2, this increase was also trending at 6mpi. C) At 18mpi, FA variance dramatically increased in G2 mice, particularly on the ipsilateral injury site. D) In contrast, the focal ipsilateral CC FA was significantly reduced at 3–18mpi in the G2 mice, and in G1 at 12 (*p* = 0.101) and 18mpi (*p* = 0.121) after juvenile CHI. E) In focal measures of the contralateral CC, FA was significantly reduced at 18mpi. F) In the focal CC measures, there was low variance in the FA at 18 mpi compared to hemispheric CC measures (see C). (Mixed effects modeling with Tukey’s multiple comparisons, *n* = 9–12/group/time point, # = *p* < 0.05, ## = *p* < 0.001, ¢ = *p* < 0.1 between G1 and Sham; * = p < 0.05, ** = p < 0.001, ┼p < 0.1 between G2 and sham).

Two independent analyses were performed, with one examining the average FA across the entire (whole) CC which was then followed by a more focal (region of interest, ROI) examination of the FA in the CC spanning the impact site. The ipsilateral hemispheric CC FA followed a similar pattern with only G1 mice reporting significant differences relative to shams at 3mpi (#p < 0.05) (Fig. 2A). In sham mice the contralateral hemispheric CC exhibited increased FA that linearly increased with age starting at 6mpi, in contrast to G1 and G2 mice, that exhibited a rapid and significant rise in FA spanning from 1 to 3mpi (*p < 0.05) (Fig. 2B). Relative to sham mice, the G2 group at 6mpi had a trending increase in FA (*p* < 0.1). We observed that the G2 group of mice had increased variance in FA in hemispheric CC measures at 18mpi compared to sham and G1 mice (Fig. 2C). This increased heterogeneity of FA within the G2 mice comparative to sham and G1 mice underlies the lack of significant findings at 18mpi despite an average FA decrease of 8.55 % in G2 mice compared to shams.

A different temporal portrait emerged when we undertook focal regional assessment centered at the impact site (region of interest, ROI, Fig. 1D). A dramatic and significant reduction in FA of the G2 mice was observed spanning the 3–18mpi (two-way ANOVA, 3 and 6mpi, *p < 0.05; 12 and 18mpi, **p < 0.001) (Fig. 2D). The ipsilateral G1 group also exhibited decrements in FA across the 12 (p-0.100) and 18mpi (p = 0.121) time points but did not reach significance. No differences were seen in the contralateral FA that exhibited a sigmodal temporal evolution (Fig. 2E) in sham, G1 or G2 except for a significant decrease (two-way ANOVA, *p < 0.05) in the G2 group at the 18mpi time point. However, in stark contrast to the variance found in the ipsilateral whole FA at 18mpi (Fig. 1C), the focal regional FA variance was markedly reduced (Fig. 2F) in the G2 mice compared to sham and G1 mice.

To further illustrate the utility of both whole and focal ROI CC measures of FA over time, we additionally plotted the percent change from shams over time (Supplementary Fig. 3A). Hemispheric CC FA analysis showed that FA increases across the entire ipsilateral CC early on but then precipitously declines after 6mpi, most notably in G2 mice. In contrast, the focal CC FA measurements from G2 mice were already below sham FA at 1mpi and continued a progressive decline over time with a 16.25 % decrease at 18mpi. Moreover, FA coefficient of variation from hemispheric CC in the G2 mice had temporal increases in variation that was contrasted by progressive declines in variation in the focal CC (Supplementary Fig. 3B). This suggests that regions distant from the injury site exhibit altered FA but large-scale microstructural impairments occur adjacent to and at the injury site.

We also examined FA metrics at the midline (center) to determine if this region exhibited increased sensitivity to potential rotational changes in the CC. We observed no overt differences in FA across the 18mpi life span between shams, G1 and G2 mice (Supplementary Fig. 4A). While the G2 group had reduced FA from 6 to 18mpi, only the final time point was significantly (*p < 0.05) reduced relative to sham mice.

Therefore, a single concussion at PND17 elicited evolving decrements in FA particularly within the more severe concussion G2 group. Further, hemispheric CC FA analysis reported global water diffusion asymmetry, while focal CC analyses can yield impactful information about alterations at the injury site, especially in ipsilateral CC. Therefore, clinical and preclinical studies should consider examination of dMRI metrics targeted to the injury site(s).

### 3.3. dMRI microstructure: radial diffusivity (RD)

**Fig. 3.**
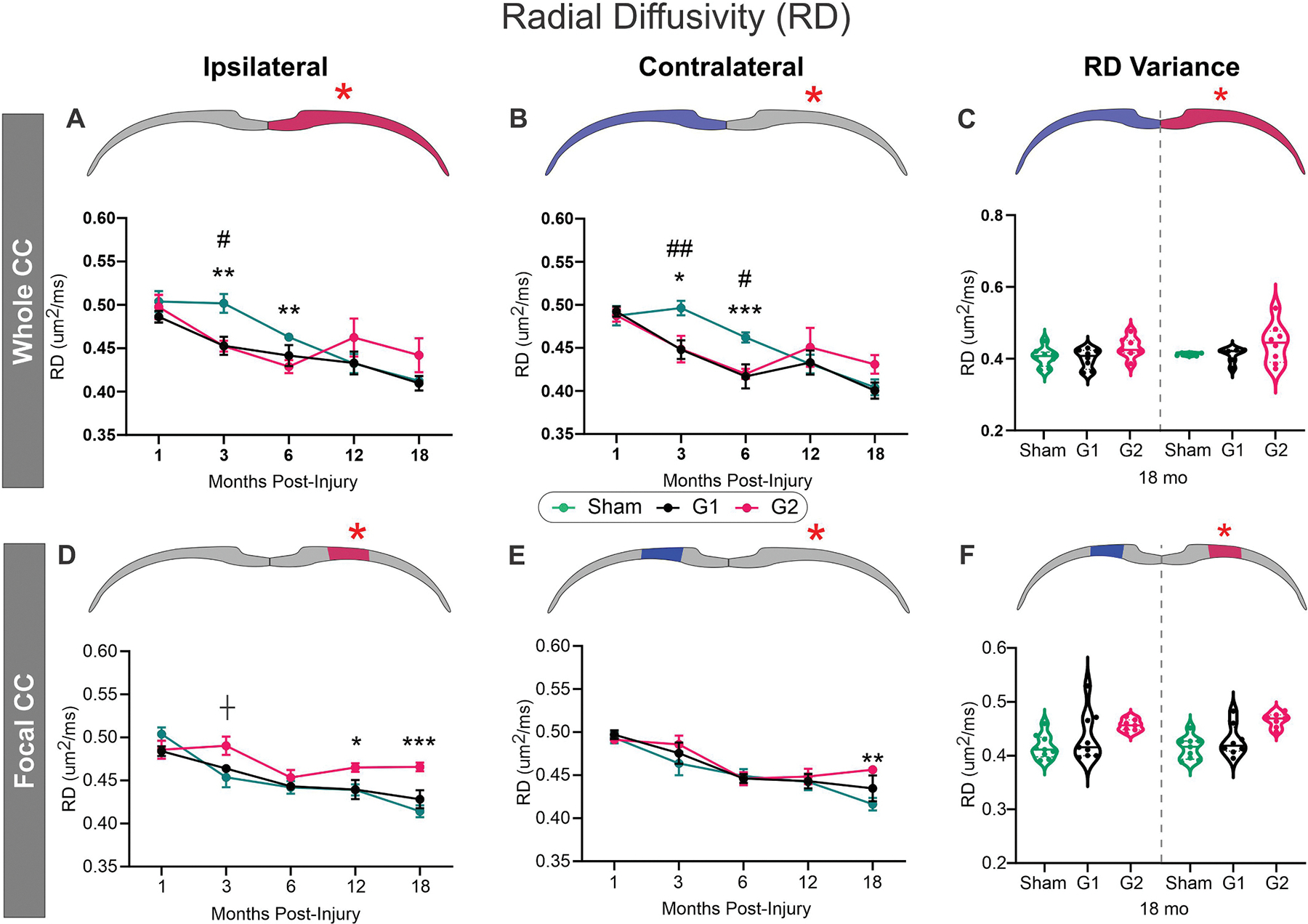
Radial diffusivity (RD) in the corpus callosum (CC) is initially decreased but elevated later in life after closed head injury (CHI) at the impact site (red asterisk). A) In the whole ipsilateral CC RD is decreased at 3 and 6mpi after juvenile CHI in both G1 and G2. B) The contralateral hemispheric CC exhibited a similar trend in both G1 and G2 but plateaued to sham levels at 12 and 18mpi. C) At 18mpi G2 mice exhibited increased variance compared to shams in the ipsilateral CC. D) In the ipsilateral focal CC a trending increase in RD was observed in G2 mice at 3mpi. At 12 and 18mpi, G2 RD was significantly elevated. E) In focal CC measures of the contralateral side, there were no overt differences except at the 18mo time point when G2 RD was elevated relative to sham mice. F) In focal CC measures the G2 mice exhibited low variance at 18mpi compared to sham and G1 mice, which also contrasts to the increased variance observed in the hemispheric whole CC RD measures (see C). (Mixed effects modeling with Tukey’s multiple comparisons, *n* = 9–12/group/time point, # *p* < 0.05, ## *p* < 0.001, between G1 and S; * p < 0.05, ** p < 0.001, *** *p* < 0.0001, ┼*p* < 0.1 between G2 and sham).

RD reports water diffusion perpendicular to the largest diffusion direction and in WM is thought to reflect axonal and myelin alterations. In hemispheric CC analysis in G1 and G2 mice, both ipsilateral (Fig. 3A) and contralateral (Fig. 3B) RD within the entire CC was significantly reduced at 3mpi (two-way ANOVA, # *p* < 0.05 and **p < 0.001) but only at 6mpi in G2 mice (two-way ANOVA, **p < 0.001) compared to shams. There were no significant differences at 12 and 18mpi despite elevated RD in G2 mice at 18mpi. Virtually identical to FA, at 18mpi there was a large increase in variance in the G2 cohort compared to sham and G1 (Fig. 3C) consistent with broad heterogenous modifications in the CC of G2 mice.

Regional focal ipsilateral CC was significantly increased in G2 at 12mpi (two-way ANOVA, *p < 0.05) and at 18mpi (***p < 0.001) (Fig. 3D). Focal CC analyses demonstrated a entirely different temporal evolution with no changes in RD until 18mpi in the contralateral CC wherein G2 RD was significantly elevated (two-way ANOVA, **p < 0.001) (Fig. 2E). RD variance in the focal CC analysis at 18mpi was reduced in G2 compared to G1 and sham mice (Fig. 3F). Similar to other dMRI metrics, RD was measured in the center CC region but was not significantly different across the 18mpi timeline between shams, G1 and G2 mice (Supplementary Fig. 4B). Post-hoc testing revealed a trending increase in RD for G1 at 1mpi (*p* = 0.071) and a significant elevation in G2 at 18mpi relative to shams.

In sum, hemispheric CC analysis showed an early (3–6mpi) decrease in RD while focal CC examination found increased RD at 12–18mpi only in the G2 cohort.

### 3.4. dMRI microstructure: mean diffusivity (MD) and axial diffusivity (AxD)

MD represents bulk water diffusion within the CC and is considered a global reporter of microstructural changes. In hemispheric CC assessments significant differences were observed in both ipsi- or contralateral CC at 3 and 6mpi in both G1 and G2 compared to sham mice (Supplementary Fig. 5A, B). The ipsilateral G1 and G2 mice showed virtually identical reductions in MD at 3 and 6mpi with reductions of ~6 % in both ipsi- and contralateral segments compared to shams. No differences were found when focal CC MD was assessed except for G2 mice having increased MD at 18mpi in ipsilateral CC compared to shams (Supplementary Fig. 5C, D).

Axial diffusivity (AxD) has been shown to reflect axonal changes within WM. Across the 3–18mpi we did not observe any significant changes in AxD in either the ipsi- or contralateral focal CC measures (Supplementary Fig. 6). However, AxD at 1mpi was reduced in G2 mice in both whole and focal CC measures. Thus, AxD results indicate reduced water diffusion at 1mpi, while MD is reduced at 3–6mpi (only in hemispheric CC) revealing temporal microstructural sensitivity to dMRI metrics after juvenile concussion.

### 3.5. Temporal evolution of variance and timeline

**Fig. 4.**
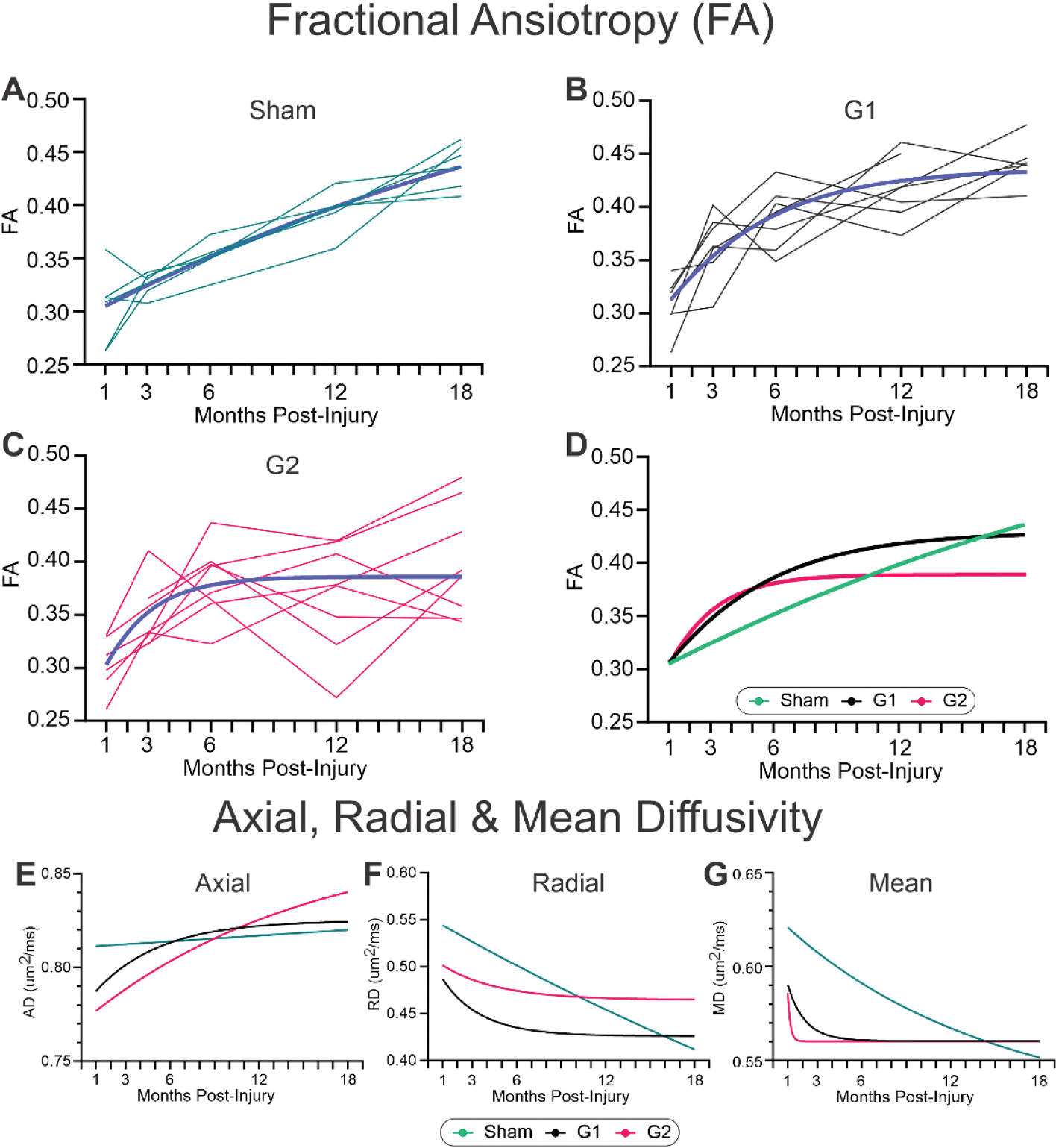
Progression of ipsilateral DTI alterations within the corpus callosum (CC) region of interest (ROI). A) Spaghetti plot of individual sham animals CC fractional anisotropy (FA) across their lifespan, with blue line representing the fitted curve, illustrating a linear relationship. B) In G1 mice, there is a curvilinear (blue line is the fitted curve, gray lines are individual mice) relationship in the FA of the CC that plateaus by 12mpi. C) Early and sustained FA increases in the CC were observed in the G2 mice that plateaued at 6mpi (blue line is the fitted curve, red lines are individual mice). D-G) The mean dMRI value from each mouse at each time point was fitted to a Gompertz growth curve using least squares fit. D) FA curve fits illustrate the remarkable temporal alterations in the FA of the CC after closed head injury (CHI). E) Axial diffusivity (AxD) was reduced early after CHI in G1 and G2 mice but only G2 mice increased progressively across 18mpi. F) Radial diffusivity (RD) of the CC linearly decreased across the lifespan in sham mice, with both G1 and G2 mice showing rapid decreases early (<3mpi) after CHI that plateaued. G) Mean diffusivity (MD) in shams exhibited a linear decrease but in CHI mice there was a very rapid decline that plateaued and was exacerbated in G2 mice. (A-C – dark blue line represents the fitted curve to all the individual mice).

We examined the temporal development of the variance we observed in dMRI metrics, focused on FA (see Fig. 2C, F), to gain a deeper insight into how individual mice progressed after concussion with age. Spaghetti plots of FA in sham mice across their lifespan illustrate remarkable homogeneity with only 1 of 10 mice having a lower FA (Fig. 4A). The G1 cohort had modest variance (average SEM 1–18mp =0.0102) with most mice clustering around the group mean (Fig. 4B). As shown in Fig. 2C and 3C, the G2 group of mice had robust variance within the CC across the whole ipsilateral CC (averaged SEM 1–18mp =0.0146, a 43.5 % increase in SEM) (Fig. 4C). An unexpected finding was the dynamic fluctuations in FA across the lifespan of some G2 mice that likely reflect individual responses to concussion and possibly differential attempts at repair within the CC.

When sham, G1 and G2 cohorts were fitted to a mono-exponential curve (Fig. 4D), the FA in shams showed a linear increase with increasing age. Both G1 and G2 in the months after a single concussive hit exhibited a rapid rise in FA that slowly plateaued to a similar FA level by 18mpi but was blunted in the G2 mice. A similar analysis was derived for the other dMRI metrics, AxD (Fig. 4E), RD (Fig. 4F) and MD (Fig. 4G). In general, shams showed linear age-related changes across all metrics. AxD was reduced early in G1 but by 3mpi plateaued to sham values which contrasts with the progressive increase in AxD across lifespan in G2. RD in G1 and G2 mice followed a virtually identical trajectory with early decreases that plateaued and were like shams at 12mpi. MD timeline was like AxD.

**Fig. 5.**
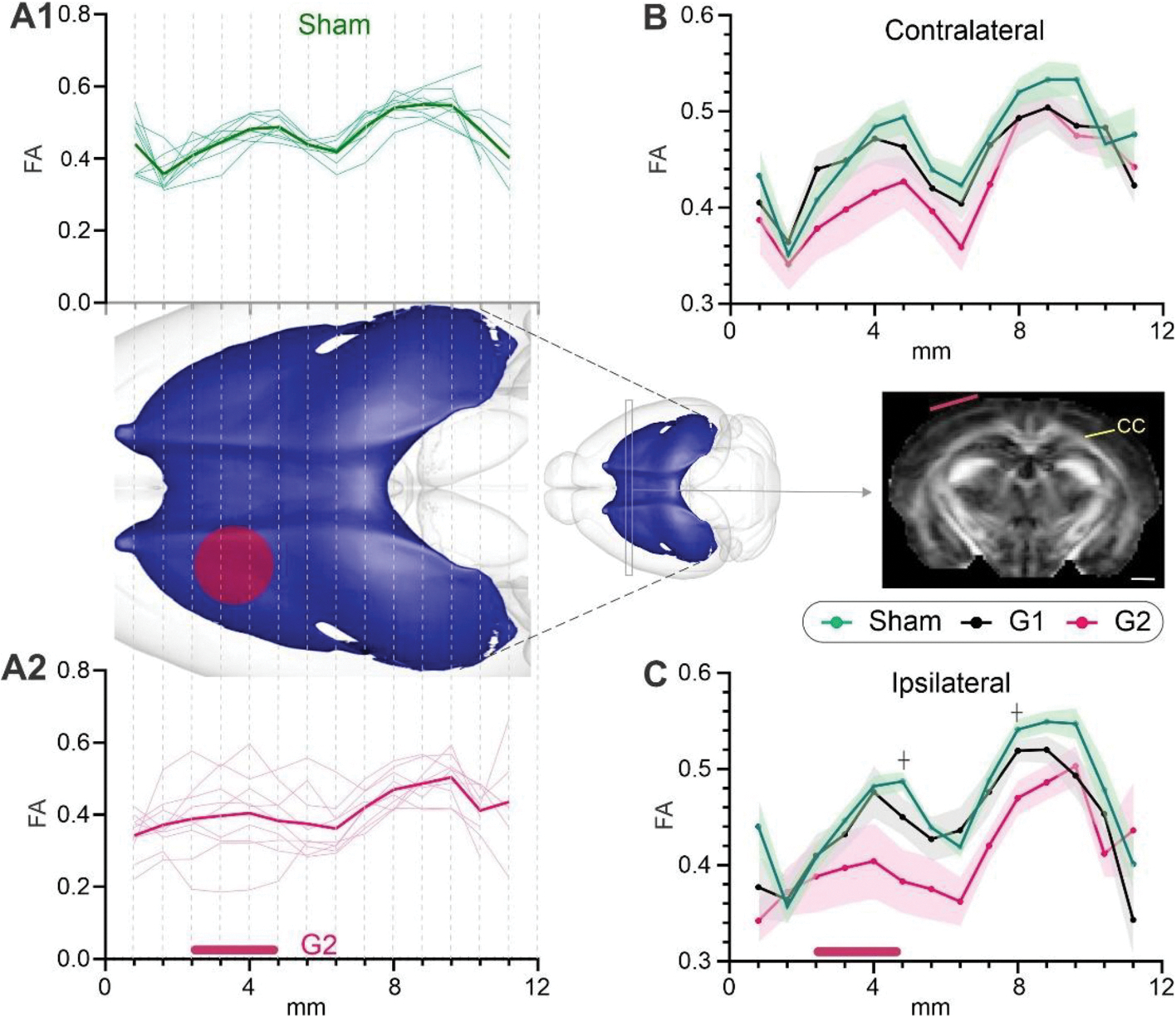
Fractional anisotropy (FA) measures illuminate altered microstructure within the corpus callosum (CC) at 18mpi. A1) FA was assessed at each MR slice (slice thickness = 0.8 mm) from anterior to posterior in ipsilateral sham (top panel). A2) Anterior to posterior FA in ipsilateral G2 mice (bottom panel). FA values were relatively homogenous in sham mice which starkly contrasted with the large variance in FA at the injury site in G2 mice. Posterior to the injury site, G2 mice exhibited more homogenous CC FA values. Insert is a 3D reconstruction of the mouse CC where the dotted lines illustrate each MRI slice. The red circle indicates the 3 mm CHI site. (MRI cal. Bar = 1 mm). B) Contralateral FA values averaged across all cohort mice for sham, G1 and G2 mice illustrate the modest reduction in FA within G2 mice. C) G2 FA values in the ipsilateral CC show a marked decrease in FA compared to G1 and sham mice. (Holm-Sidak multiple *t*-tests, *n* = 9/group, ┼ *p* < 0.1 between G2 and Sham. Maroon bar in graphs illustrate location of the CHI.

Whilst the global CC metrics and their variance are informative, we wished to determine where along the CC anterior-posterior extent the variance was derived; in other words, was the variance global or more focal in nature. For these analyses we extracted from each MRI slice the FA along the entire ipsilateral or contralateral CC at 18mpi (Fig. 5) which were then plotted in an anterior-posterior progression as illustrated in Fig. 5A for the ipsilateral CC. In sham mice (Fig. 5A1) there was uniform variance across the anterior-posterior extent of the CC in contrast to the robust increased variance in anterior CC at the level of the impact site (red circle on 3D CC reconstruction – center) of G2 mice (Fig. 5A2). The variance was also increased in more posterior aspects of G2 mice. We next plotted the average FA in the CC from the contralateral (Fig. 5B) and the ipsilateral (Fig. 5C) hemispheres but found no significant deviations from shams despite the large FA decrease seen at the impact site in G2 mice. Despite the large FA decreases, the lack of significant changes was attributed to the increased G2 variance.

### 3.6. Astrocyte and microglial morphology after concussion

From our longitudinal MRI cohort, we extracted multiple G2 CHI and sham mice at selected time points for histological evaluation of inflammatory markers. Our significant findings from the focal CC in FA and RD at 12 and 18mpi prompted us to assess if there were any astroglial perturbations at time points relative to the 1mpi. At 12mpi, contralateral and ipsilateral CC of G2 CHI mice glial fibrillary astrocytic protein (GFAP) staining (Supplementary Fig. 7A, B) was present at 1 and 12mpi with no significant difference in staining intensities (Supplementary Fig. 7 D) compared to sham mice. We then performed skeleton analysis on astrocytes from the ipsilateral CC to quantify different cell shape features. While there were no significant differences in total astrocyte process length between G2 and sham groups at the 1mpi time point (*p* = 0.622), there was a significant decrease (repeated measures t-test, **p < 0.01) at 12mpi (Supplementary Fig. 7C). At 12mpi, sham astrocytes had highly branched skeletal morphology whereas G2 astrocytes had significantly decreased branches and junctions (Supplementary Fig. 7B, C, unpaired *t*-test, *p < 0.05). No significant differences were observed at 1mpi between G2 and sham groups in, mean gray values (Supplementary Fig. 7D), number of branches (unpaired t-test, *p* = 0.619, Supplementary Fig. 7E) or junctions (*p* = 0.559, Supplementary Fig. 7F).

**Fig. 6.**
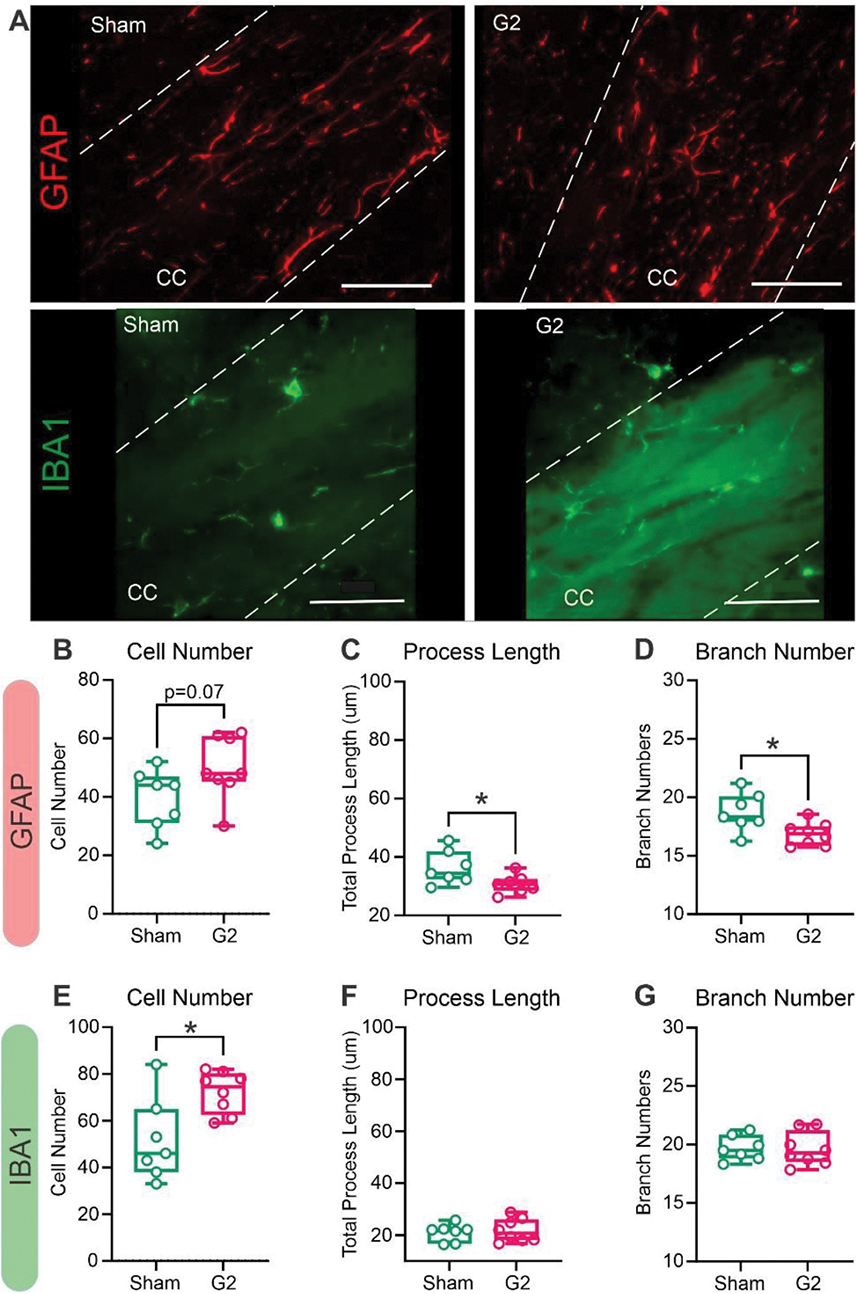
Astrocyte and microglia morphological features at 18mpi within the corpus callosum (CC). A) Representative GFAP-positive astrocytes and IBA1-postive microglia within the CC at 18mpi in sham (left) and after juvenile CHI (right). B) Astrocyte cell numbers were elevated but not significantly different at 18mpi. C) Astrocyte process lengths were significantly decreased, consistent with a more “amoeboid”-like shape. D) Similarly, the number of astrocyte branches also decreased at 18mpi. E) The number of microglial cells within the CC were significantly increased. F) Microglial processes lengths were unaltered at 18mpi. G) Branching of microglia were also not significantly changed between G2 and sham mice. (*t*-test, n = 7–8/group, * *p* < 0.05). Scale bar =50 μm.

We undertook analysis of GFAP morphology at 18mpi with a larger number of replicates (*n* = 7–8 mice/group) as this was the final time point in our study. There were increased cell numbers with reduced branching in G2 mice (Fig. 6A). Quantification of GFAP cell numbers exhibited a trending increase (*p* = 0.07) at 18mpi (Fig. 6B). We observed significant decreases in astrocytic morphological features including process length (* *p* < 0.05) (Fig. 6C) and number of branches (* p < 0.05) (Fig. 6D). Similarly, significant reductions in GFAP number of junctions (* *p* < 0.05) (data not shown) and in total branch lengths (* p < 0.05) (data not shown). These results at 18mpi, are consistent with increased “ameboid”-like morphology of astrocytes in the CC after CHI.

Long-term inflammatory responses after TBI have been reported by our group and others (Obenaus et al., 2023; Chen et al., 2023). Given the phenotypic changes in astrocytes, we then investigated if microglia (via IBA1 immunohistochemistry) within the ipsilateral CC exhibited an activated morphology characteristic of inflammatory states. There were no differences in staining intensity at 12mpi between sham and G2 mice in either the contralateral or ipsilateral CC (Supplementary Fig. 8A). However, the morphology of G2 microglial cells was altered compared to sham mice with a considerable decrease in cellular arborization (Supplementary Fig. 8B). We quantified these changes using fractal analysis and found that the span ratio of microglial cells was significantly decreased in G2 compared to sham mice in the CC (unpaired t-test, *p < 0.05) with a non-significant decrease in cell density which represents the cell size (Supplementary Fig. 8C, *p* = 0.474). Moreover, the decrease in span ratio coincided with increased cell circularity (unpaired t-test, *p < 0.05) suggesting an activated microglial phenotype (Supplementary Fig. 8C). No significant differences were detected at 12mpi in microglial cell complexity (fractal dimension), cell area and perimeter between sham and G2 group (Supplementary Fig. 8D). Skeleton analysis performed in ipsilateral CC at 12mpi did not show differences in the number of branches or junctions, nor in total process length in microglia (Supplementary Fig. 8E), nor mean gray values (Supplementary Fig. 8F).

Microglial responses at 18mpi exhibited significantly increased number of cells in G2 CHI compared to shams (* *p* < 0.05) (Fig. 6A, E). Unlike GFAP-positive astrocytes, microglia did not show any significant changes in process lengths (Fig. 6F), number of branches (Fig. 6G) nor number of junctions (data not shown). Taken together, both astrocytes and microglial cells within the CC at 12 and 18mpi exhibited increased cell numbers with reduced morphological features evident only in astrocytes. NF200 cell staining density was not altered after CHI at either 1 or 12mpi (Supplementary Figs. 2, 9).

### 3.7. Relationship between dMRI and astrocytes and microglia

**Fig. 7.**
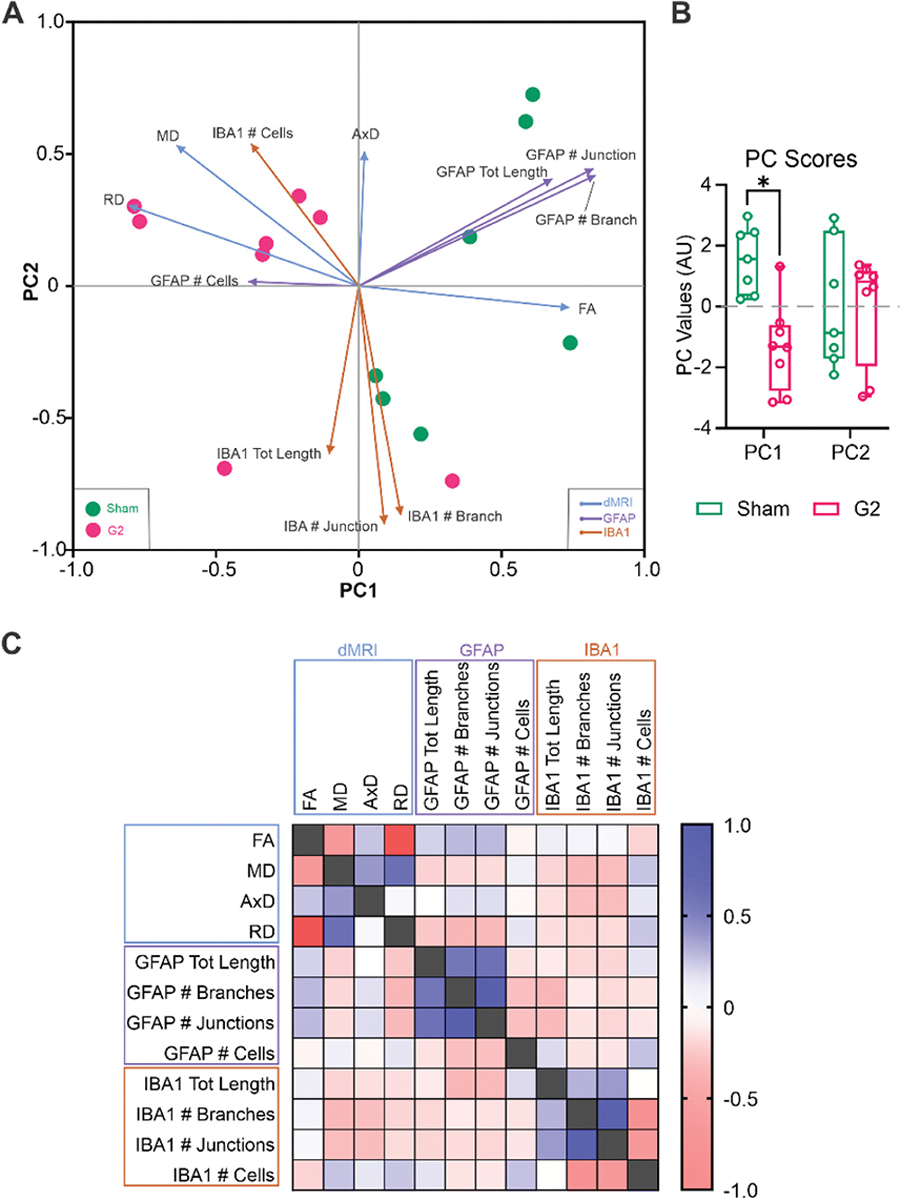
Relationship between dMRI measures and glial cell features. A) Principal component analyses of dMRI metrics (FA, MD, RD and AxD) revealed predominate positive correlations to astrocytes (GFAP) but were negatively correlated to microglial features (IBA1). B) Comparison of PC1 scores found a significant decrease in G2 mice compared to shams (Mann-Whitney test, * p < 0.05). C) Pearson correlation matrix for dMRI metrics versus astrocyte and microglial cellular features, where astrocytes were modestly positively correlated with FA but weakly positively correlated with AxD. RD and MD were negatively correlated with microglial features. Supplementary Table 3 has *p*-values associated with the correlation matrix.

To further explore the influence of glial cells on dMRI metrics on the final timepoint we undertook principal component analysis (PCA) to define the similarities between individuals from the dMRI values and glia immunolabeling quantification. Two dimensions (PC1 and PC2) described a total of 60.04 % of proportion of variance between the individuals. PC1 score was positively correlated with FA, GFAP-positive astrocyte morphological parameters and negatively correlated with numbers of GFAP-positive astrocytes, MD and RD (Fig. 7A). PC2 score was positively correlated with AxD and negatively correlated with morphological parameters from IBA1 staining (Fig. 7A). PC1 was able to differentiate between shams and G2 CHI mice but not PC2 (Fig. 7A) which was further confirmed in evaluating the PC scores (*p < 0.05, Mann-Whitney comparison of ranks, Fig. 7B).

Correlational analysis examined the influence of glial cell changes on dMRI. Pearsons correlations, for the most part, highlighted moderate to strong negative correlations with MD, AxD, and RD, but positive correlations with FA (Fig. 7C). Astrocyte features were modestly positively correlated with FA and weakly positively correlated with AxD (Fig. 7C). RD and MD were negatively correlated with astrocyte features. Broadly, these analyses may suggest that microglial features modulate MD and RD, whereas astrocytes may contribute more greatly to AxD and FA.

## 4. Discussion

Concussion injuries continue to figure prominently in athletic, military, and civilian populations. Studies have alluded to long-term decrements in concussion individuals, including learning and memory, and neuropsychiatric disturbances (de Neeling et al., 2023). Critical to understanding the sequela that emerges after concussion are how WM is modified over time. While progress has been made, long-term pre-clinical studies focused on WM are lacking. To address this gap, we developed a concussion model in juvenile mice implementing a CHI protocol. Sequential dMRI from 1 to 18mpi was performed and analyses focused on the largest WM tract in the mouse, the corpus callosum (CC). Our novel time course neuroimaging study found: 1) whole hemispheric CC analysis in G2 mice identified decreased AxD at 1mpi followed by elevated FA and decreased RD at 3–6mpi which mirrored the MD decrements, 2) Focal examination of dMRI metrics at the concussion site reported progressive decrements in FA from 3mpi onwards, with RD being elevated only at 12–18mpi. AxD was decreased at 1mpi followed by decreased RD at 3–6mpi, 3) FA and RD from hemispheric CC analysis across injury groups exhibited increased heterogeneity with increasing severity of concussion, which was predominately localized to the concussion site, 4) Astrocyte and microglial morphology exhibited a trend for reactive phenotypes at 12mpi but not 1mpi, and 5) At 18mpi only GFAP-positive astrocyte morphologies contributed to dMRI changes in CC. In sum, our findings suggest a progressive temporal vulnerability to the CC after a single juvenile concussion. We also report that a more severe concussion elicits enduring WM alterations across the lifespan.

The developing brain, in particular WM, is especially vulnerable to concussion and traumatic brain injury. As reviewed by Semple and colleagues, WM myelination, synaptogenesis and pruning all are active during adolescence (Semple et al., 2013). Our recently developed mouse concussion model induces injury at post-natal day 17 (PND17) and includes a rotational aspect with early MRI modifications in the CC (Rodriguez-Grande et al., 2018). We also found that two different concussive severities (G1, G2) resulted in similar perturbations, both on MRI and histopathology, but G2 reported exacerbated CC alterations. We undertook a temporal study spanning 1–18mpi in a cohort of mice that received sham, G1 or G2 concussions using dMRI with glial histopathology assessments. This study is the first to temporally evaluate concussion across the mouse lifespan. A recent adult mouse study found regional CC FA decrements and volumes at both 6 and 12mo but only in repeated mild closed head injury (rmCHI) but not in single CHI mice (Moro et al., 2023). In our studies we found significant CC FA changes in G2 mice as early as 3mpi and in G1 mice at 12 and 18mpi when regional CC analyses were performed. When hemispheric CC analysis was performed, only the 3 and 6mpi had group-wise differences, due in part to the heterogeneity of FA changes across the entire hemispheric CC. The differences between Moro et al. and our findings can be attributed to concussion model differences; our model explicitly has a rotational component and is at a younger age which potentially increases vulnerability due to WM perturbations during development (Rodriguez-Grande et al., 2018).

Several studies have reported neuroimaging alterations within the CC at early chronic epochs in more severe TBI models. Parent and colleagues noted CC FA reduced at 2mpi in rats after a diffuse fluid percussion injury (FPI) during late adolescence (PND31) (Parent et al., 2019). In an open skull cortical contusion model (CCI) mild TBI model we found reduced MD (but not FA) at 2mpi which correlated to increased myelin components in anterior CC (Wendel et al., 2018). In a young adult rat rmTBI model there was reduced FA in the CC at 2.5mpi with no attendant changes in AxD, RD or MD (Yu et al., 2017). Another study in a moderate single TBI (CCI) in PND 17 days old rats reported decreased CC thickness at 2mpi with increased myelin basic protein and loss of conductivity in CC fibers (Ajao et al., 2012). Similarly, using CCI model in adult rat at 1mpi there were widespread FA reductions in the CC that were evident as early as 1wk after injury, in part due to the more severe nature of the TBI model (Harris et al., 2016). Thus, despite differences in models utilized, regions of interest, and methods of analyses, there is strong evidence for early and sustained vulnerability to concussive injury, particularly to the CC. Indeed, many studies have noted cognitive deficiencies in rodent concussion models even early after injury suggesting that a linkage between behavior and WM integrity after concussion (Moro et al., 2023; Obenaus et al., 2023; Yu et al., 2017; Ajao et al., 2012).

A novel aspect of the present study is that we were able to undertake a longitudinal dMRI study within the same mice across 18mpi. These longitudinal studies are difficult, expensive and time consuming, but they provide an exceptional perspective of how WM in each subject (mouse) progresses across their lifespan. One facet we observed in our temporal studies was the increased variance in dMRI metrics, particularly in the G2 group (Fig. 4). Sham and G1 mice across their lifespan had a relatively consistent variance whilst the G2 group had considerable inter-mouse variability in the FA across their lifespan. Indeed, considerable variance has also been noted in adult and pediatric clinical populations (Ware et al., 2022; Oehr et al., 2021; Palacios et al., 2022; Stillo et al., 2021). One suggestion to limit this variability is to use symmetry measures to blunt TBI-associated effects on FA (Vakhtin et al., 2020). Alternatively, this variance has been suggested to represent individual resilience and may relate to recovery (or lack) after TBI when examining WM tracts (Cai et al., 2022; Schmidt et al., 2021). Future work in animal models of concussion should pursue the linkage between variance in dMRI metrics and behavioral outcomes. We have previously reported associations between FA in the hippocampus and cognitive tests (Obenaus et al., 2023).

Not all dMRI metrics are created equal nor report the same microstructural details longitudinally. In our study we observed that FA was the most robust reporter of CC alterations over time, particularly in the focal regional CC analyses (Fig. 2). At earlier time points, AxD, RD and MD selectively model changes in the WM microstructure, whereas FA increasingly identifies abnormalities in regional CC metrics. Several reviews encompassing dMRI and TBI have also summarized that FA is indeed a sensitive potential biomarker (Delouche et al., 2016; Turner et al., 2021), although there are conflicting reports as well. Monitoring FA, for example at the injury site, could provide the basis for clinical diagnostics related to progression and recovery as well as assessing future therapeutics. Clinically, FA (as well as other metrics) have been shown significant sensitivity in mTBI patients (Palacios et al., 2022). Our findings and those from the literature advocate for monitoring FA closely in mTBI and concussion patients at short- and long-term after brain injury.

The physiological, cellular and molecular alterations that underlie the progressive changes in the CC after a concussion remain to be fully elucidated, although ultrastructural studies have demonstrated cellular and functional decrements over time (Marion et al., 2018). A primary focus has been the inflammatory cascade that ensues after concussion particularly in the acute and sub-acute epochs (Verboon et al., 2021). One year after rmTBI in the CC histopathological evidence of axonal injury is evident, with a 51 % reduction in myelin, increased astrocytes (116 %), and microglia (69 %) (Moro et al., 2023). In our study an interesting finding was that the anterior CC was more affected than posterior regions. This is perhaps not overtly surprising as these alterations were found at the concussion site and possibly reflects head rotation in our concussion model. We previously reported that at 1, 7 and 30d post injury there was increased glial fibrillary acidic protein (GFAP; astrocytes) staining in this concussion model within the CC (Rodriguez-Grande et al., 2018). In our current study we did not observe any overt astroglia staining at 1mpi, 12mpi but by 18mpi in CC there was a significant reduction in astrocyte branching and process lengths suggesting reduced spatial coverage. Another study showed process shortening in CC fibrous astrocytes in response to axonal injury (stab wound) followed by process growth and glial scar formation (Sun et al., 2010). The functional role of these morphological changes in astrocytes in response to brain injury and whether these changes are linked to a specific inflammatory state remain unknown. The modulation of myelin dysfunction/repair by astrocytes has not been studied in TBI but research in multiple sclerosis models have observed a dynamic communication between glia and suggests an avenue of future research (Schroder et al., 2023).

Early and acute microglial responses in TBI and concussion have been well documented in our studies (Table 1) and by others (Jullienne et al., 2020; Mohamed et al., 2020; Shitaka et al., 2011; Taib et al., 2017). IBA1 immunolabeling in our current study showed a more ameboid microglial cell morphology with reduced span ratio and increased circularity in the more severe G2 concussion mice. We did not find increased microglial density in our concussion study at 12mpi, but others have reported increased CC microglial density at the same time point which is likely due to their repeated TBI model (Moro et al., 2023). In a study of human TBI there was evidence of neuroinflammation up to 17 yrs. post injury (Johnson et al., 2013). Microglia that contain lipofuscin exhibited increased phagocytic activity in aged compared to young mice and TBI in old mice (18mo) elicited a robust phagocytic response in a sub-population of microglia despite no differences in the number of microglia (Ritzel et al., 2023). Despite the paucity of studies evaluating the long-term microglial response after concussion and TBI, there appears to be ongoing microglial inflammation years after injury, as highlighted in this recent review (Wangler and Godbout, 2023). Modulation of oligodendrocytes by microglia can impact myelination after TBI as a potential mechanism to improve outcomes (Song et al., 2022).

Future dissection of FA metrics could point to underlying cellularity after concussion. One such approach is use of linear Westin anisotropic metrics which describe how spindle-shaped the FA ellipsoid is, while the planar metric portrays how the disc shaped it is, and the spherical component explains the sphericity of the ellipsoid (Curran et al., 2016). These metrics have been used previously to characterize WM alterations and could yield additional insights (Chang et al., 2017). Diffusion MRI can also be combined with positron-emission tomography (PET) for microglial markers (Masdeu et al., 2022). An example of combinatory imaging in severe TBI was reported by Missault and colleagues (Missault et al., 2019), where they showed increased inflammation (PET) and acute reductions in FA (MRI) that were correlated with behavioral decrements. Future combinatory studies should also evaluate fluid biomarkers for relationships between FA, PET, and inflammatory biomarkers.

There are several limitations in the current study. This longitudinal cohort was limited to males and ideally should have included female mice as well. Moro found no sex differences in their long-term rmTBI studies (Moro et al., 2023). While sex differences have been reported in TBI (see review (Biegon, 2021)), no overt consensus has yet emerged, particularly with regard to adolescent concussions. A caveat of our study is designation of PND17 mice defined as human adolescents. There is considerable literature describing the brain and WM (myelination) development in both humans and mice, but linkage has been difficult (Buyanova and Arsalidou, 2021; Cottam et al., 2024; Lebel and Deoni, 2018; Tamnes et al., 2018). We have used a holistic approach as reviewed by Semple and colleagues in defining our developmental epoch for concussion induction (Semple et al., 2013). The current study would be strengthened with a larger longitudinal cohort that would allow cross-sectional extraction of sufficient mice at each time point for immunohistochemical and molecular quantifications. In addition, new emerging advanced dMRI approaches such as multi-shell dMRI, can provide fresh non-invasive insights into the pathophysiology of WM (Lima Santos et al., 2022; McCunn et al., 2021).

## 5. Conclusions

The clinical and neuropsychological consequences of adolescent concussion are starting to emerge. However, there is a gap in preclinical literature with a lack of long-term assessments using clinical metrics, such as dMRI, to probe WM vulnerability after adolescent brain injury. In our study, we provide the first long-term evidence of altered WM microstructure, where early (1mpi) AxD reminiscent of axonal damage is followed by myelin decrements as reported by RD. FA was altered as early as 3mpi and continued to consistently be reduced across the lifespan of the concussed mouse up to 18mpi. Brain injury in juvenile mice had a long-term impact on astrocytes which exhibited decreased complex morphology. Microglial cellular morphology in WM was consistent with an increased phagocytic/inflammatory phenotype. Another key finding was that the more severe concussion modality exhibited increased variability within the CC and may represent individual mice that might be attempting endogenous recovery of WM. Future work should utilize this information to guide therapeutic interventions. In summary, we demonstrate progressive and enduring changes within the mouse CC across its lifespan following a single concussion overlying the somatosensory cortex. This study also highlights the paucity of longitudinal studies in both sexes and additional efforts should be undertaken to fill this gap in knowledge.

## Supplementary Material

Obenaus 2025 Supplementary Material

Appendix A. Supplementary data

Supplementary data to this article can be found online at https://doi.org/10.1016/j.expneurol.2025.115455.

## Figures and Tables

**Table 1:** Recent publications and findings related to closed head injury with long-term disabilities (CHILD).

First Author, PMID	Outcome measurements							Timepoints after Injury
	Sex	Behavior	MRI	Brain regions (ROI)	Histology	Physiological measurements	Other	

Rodriguez-Grande, 29665077	Males	Foot-fault, T-Maze, open field	T2 and DTI with FA	Corpus callosum	IgG, AQP4, GFAP, NF200, MBP	–	Kinematics, head rotation	1,3,7 & 30d
Clément, 31670865	Males	–	T2 and DTI	Dentate gyrus, Somatosensory cortex, amygdala and infralimbic area	NeuN, GFAP, Nestin	–	–	1,3,7 & 30d
Ichkova, 32442681	Males	Open field	T2	Somatosensory cortex	IgG, Tomato-Lectin, NeuN and NF200	Ex-vivo vascular coupling, brain Oxy-hemo photoacoustic and EEG	–	6 h, 1,3,7,30d
Leyba, 36625322	Males	Open field and novel object recognition	–	–	–	Brain Oxy-hemo photoacoustic	Cardiac Oxy-hemo photoacoustic	4 h, 7, 30, 90, 240d
Obenaus, 36859364	Males	Open field, elevated plus maze, sucrose preference, Morris water maze, novel location tests	T2, DTI	Substantia Innominate (SI), Nucleus basalis, Medial septal nucleus, hippocampal subregions (CA1, CA2 andCA3) and dentate gyrus	NeuN, APP, NF200, GFAP, IBA1, tomato-lectin, AQP4	–	–	2, 6 and 12 months
Dubois, 39992806	Males & Females	Time to recover	–	Somatosensory cortex	IgG, GFAP	–	Detailed protocol	1d
Badaut, 38352553	Males	Open field, elevated plus, novel object recognition, hot-plate, Beam walk, rotarod test	T2, DTI, (tractography)	Somatosensory cortex	GAD67, Paravalbumin neurons	Ca^2+^ neuronal activity Mini scope	Pharmaco-treatment and classification algorithm (AI neural network)	3,6,9 and 12 months
Current manuscript	Males		T2, DTI	Corpus callosum	NF200, GFAP, IBA1	–	–	1,3,6,12 and 18 months

## Data Availability

Data will be made available on request.

## Abbreviations:

ANOVA: analysis of variance
AxD: Axial diffusivity
CC: corpus callosum
CHI: closed head injury
Cg: cingulum bundle
DAPI: 4′,6-diamidino-2-phenylindole
D_B_: fractal dimension
dMRI: diffusion magnetic resonance imaging
DTI: diffusion tensor imaging
FA: fractional anisotropy
FFT: Fast Fourier transform
FOV: field of view
G1: grade 1 of closed head injury
G2: grade 2 of closed head injury
GFAP: glial fibrillary acidic protein
IBA1: Ionized calcium-binding adaptor molecule 1
IHC: immunohistochemistry
IQR: interquartile range
MD: mean diffusivity
mpi: months post injury
MRI: magnetic resonance imaging
mTBI: mild traumatic brain injury
NF200: neurofilament-200
PBS: phosphate-buffered saline
PET: positron-emission tomography
PND: postnatal day
rmTBI: repeated mild traumatic brain injury
ROI: region of interest
RD: radial diffusivity
T2WI: T2-weighted imaging, also T2
WM: white matter
