# Supplementary material for: Progressive lifespan modifications in the corpus callosum following a single concussion in juvenile male mice monitored by diffusion MRI
Source: Exp Neurol. Author manuscript; Available in PMC 2026 Feb 11. (PMC12893616; doi:10.1016/j.expneurol.2025.115455)
Supplement: Obenaus 2025 Supplementary Material [file NIHMS2138380-supplement-Obenaus_2025_Supplementary_Material.docx]

**Progressive lifespan modifications in the corpus callosum following a single juvenile concussion in male mice monitored by diffusion MRI**

Andre Obenaus^a*^, Brenda P. Noarbe^a^, Jeong Bin Lee^b^, Polina E. Panchenko^c^, Fang Tong^c^, Sean D. Noarbe^a^, Claire Bottini^c^, Yu Chiao Lee^a^, and Jerome Badaut^c, b, d^

^a^Department of Pediatrics, School of Medicine, University of California Irvine, Irvine, CA, USA,

^b^Basic Science Department, Loma Linda University School of Medicine, Loma Linda, CA, US,

^c^CNRS UMR 5536 RMSB-University of Bordeaux, Bordeaux, France

^d^CNRS UMR 7372 CEBC-La Rochelle University, Villiers-en-Bois, France

Running Title: Concussion modifies corpus callosum across lifespan

*** Corresponding Author and Current Address:**

Andre Obenaus, Ph.D.

Division of Biomedical Sciences

School of Medicine

University of California Riverside

SOM Research Bldg, Rm 207

Riverside, CA 92521

Office 951-827-0735

aobenaus@medsch.ucr.edu

**Supplementary Figures and Tables**

**Supplementary Table 1A: Number of mice at each time point**

| Time point (mpi) | Sham | G1 | G2 |
| --- | --- | --- | --- |
| 1 | 12 | 13 | 10 |
| 3 | 9 | 11 | 10 |
| 6 | 10 | 11 | 11 |
| 12 | 9 | 11 | 10 |
| 18 | 9 | 8 | 10 |

mpi = months post injury

**Supplementary Table 1B: Cerebrum volumes across lifespan**

| Time point (mpi) | Sham | G1 | G2 |
| --- | --- | --- | --- |
| 1 | 262.08 ± 6.85 | 311.50 ± 9.42 | 262.77 ± 7.55 |
| 3 | 287.24 ± 6.61 | 303.33 ± 4.13 | 297.60 ± 4.55 |
| 6 | 330.30 ± 4.71 | 319.26 ± 3.27 | 321.09 ± 2.60 |
| 12 | 324.47 ± 3.36 | 324.37 ± 2.45 | 317.19 ± 4.19 |
| 18 | 337.28 ± 4.07 | 333.62 ± 3.13 | 329.54 ± 3.91 |

mpi = months post injury


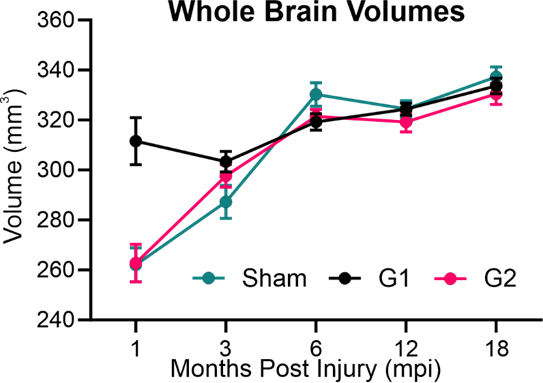


**Supplementary Table 1B Graph**.

**Supplementary Table 2: Corpus Callosum (CC) volumes (mm^3^)**

*(Figure 1F in tabular form)*

**Ipsilateral CC**

| Time point (mpi) | Sham | G1 | G2 | Change G1 | Change G2 |
| --- | --- | --- | --- | --- | --- |
| 1 | 3.87 ± 0.36 | 3.80 ± 0.28 | 3.77 ± 0.22 | -1.80% | -2.65% |
| 3 | 3.86 ± 0.30 | 4.10 ± 0.21 | 3.99 ± 0.17 | 6.17% | 3.29% |
| 6 | 4.19 ± 0.44± | 4.08 ± 0.20 | 4.03 ± 0.23 | -2.68% | -3.82% |
| 12 | 4.21 ± 0.31 | 4.03 ± 0.36 | **3.88 ± 0.09*** | -4.23% | -7.69% |
| 18 | 4.06 ± 0.37 | 4.13 ± 0.22 | 4.10 ± 0.22 | 1.80% | 0.98% |

* p<0.05 from Sham

| Time point (mpi) | Sham | G1 | G2 | Change G1 | Change G2 |
| --- | --- | --- | --- | --- | --- |
| 1 | 3.62 ± 0.14 | 3.75 ± 0.31 | 3.68 ± 0.20 | 3.66% | 1.60% |
| 3 | 3.76 ± 0.27 | **4.08 ± 0.27*** | 3.97 ± 0.27 | 8.54% | 5.53% |
| 6 | 4.14 ± 0.46 | 4.06 ± 0.25 | 3.93 ± 0.19 | -2.00% | -5.08% |
| 12 | 4.04 ± 0.03 | 4.09 ± 0.35 | **3.82 ± 0.12***** | 1.28% | -5.43% |
| 18 | 4.00 ± 0.38 | 4.11 ± 0.26 | 4.01 ± 0.39 | 2.67% | 0.18% |

**Contralateral CC**

* p<0.05 from Sham

*** p<0.0001 from Sham

**Supplementary Table 3: Significance values for Pearsons Correlations (see Figure 7)**

Red = p<0.05, Yellow p<0.01

**
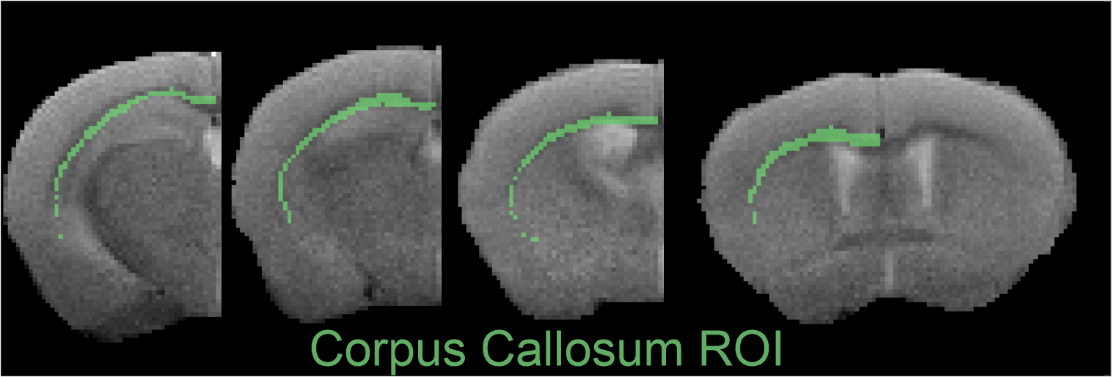
**

**Supplementary Figure 1. Exemplar CC regions of interest (ROI).** CC ROIs derived from a modified Australian Mouse Brain Consortium (AMBC) atlas, see (Ullmann, Watson et al. 2013).


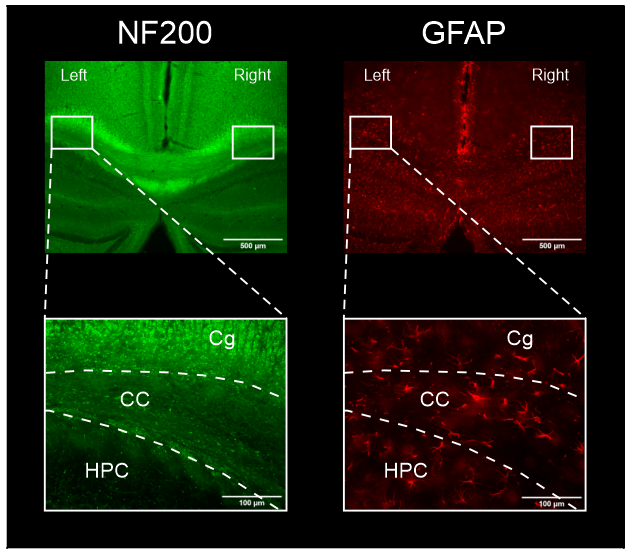


**Supplementary Figure 2. Regions of interest (ROI) in lateral corpus callosum (CC) used for quantitative analyses.** Images of corpus callosum were acquired from immunofluorescent histological coronal sections with 4X (top) and 20X objectives (bottom) in both hemispheres for each animal. Sham mouse at 12mpi time point is represented on this figure. Approximate imaged regions are indicated by white rectangles. Corpus callosum (CC) ROI used for quantitative analyses was manually delineated first on NF200 images (left) and then copied on GFAP image (right). Scale bars: 500 µm for 4X magnification and 100 µm for 20X magnification. Cg – cingulum bundle, HPC – hippocampus.


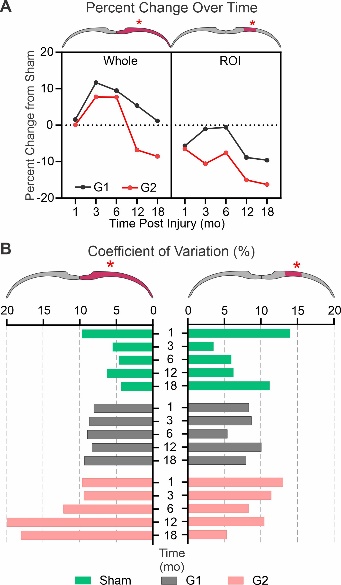


**Supplementary Figure 3. Fractional Anisotropy (FA) percent change and variance over time post injury.** A) Temporal ipsilateral corpus callosum (whole CC) changes in FA relative to shams illustrate the increased sensitivity to altered diffusion MRI (dMRI) microstructure, where focal analysis reports continued decline in FA in G2 mice. At later time points whole CC analysis also report declines but only at time points >12mo post injury (mpi). B) Additional analysis of the variance in FA at each time point and across groups shows that with the whole CC analysis, that broadly, there is a temporal increase in variance with time. In contrast, the focal ROI analysis has a temporal decline in variance. Both whole and focal CC analysis provide detailed FA information, but we posit that focal ROI early after injury may be more efficacious in early monitoring of concussion subjects.


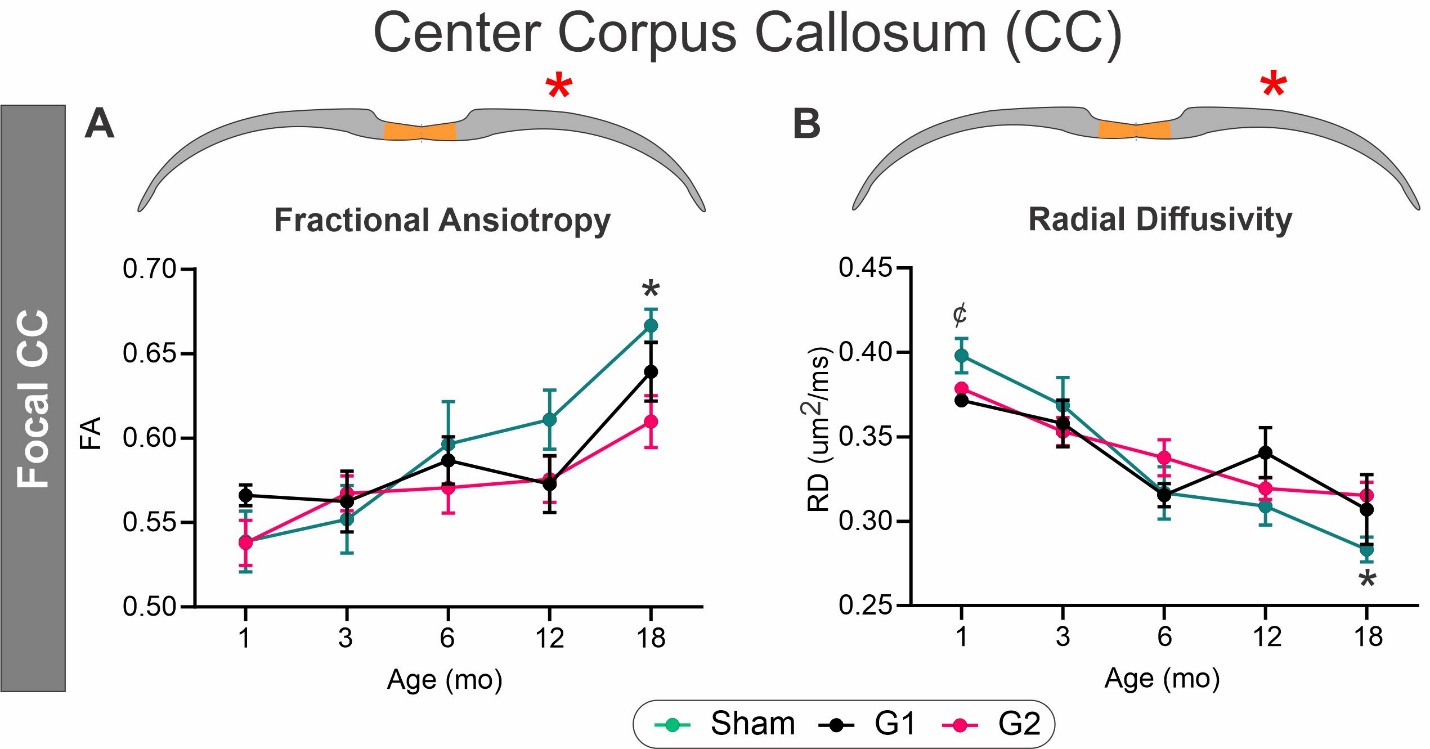


**Supplementary Figure 4. dMRI metrics from the midline (center) corpus callosum.** A) Fractional anisotropy of the center CC region of interest was significantly reduced in G2 mice at 18mpi (* p<0.05). B) Inversely, radial diffusivity (RD) was significantly increased in G2 mice at 18mpi (* p<0.05). G1 and Shams exhibited a trending difference at 1mpi (¢ p<0.071).


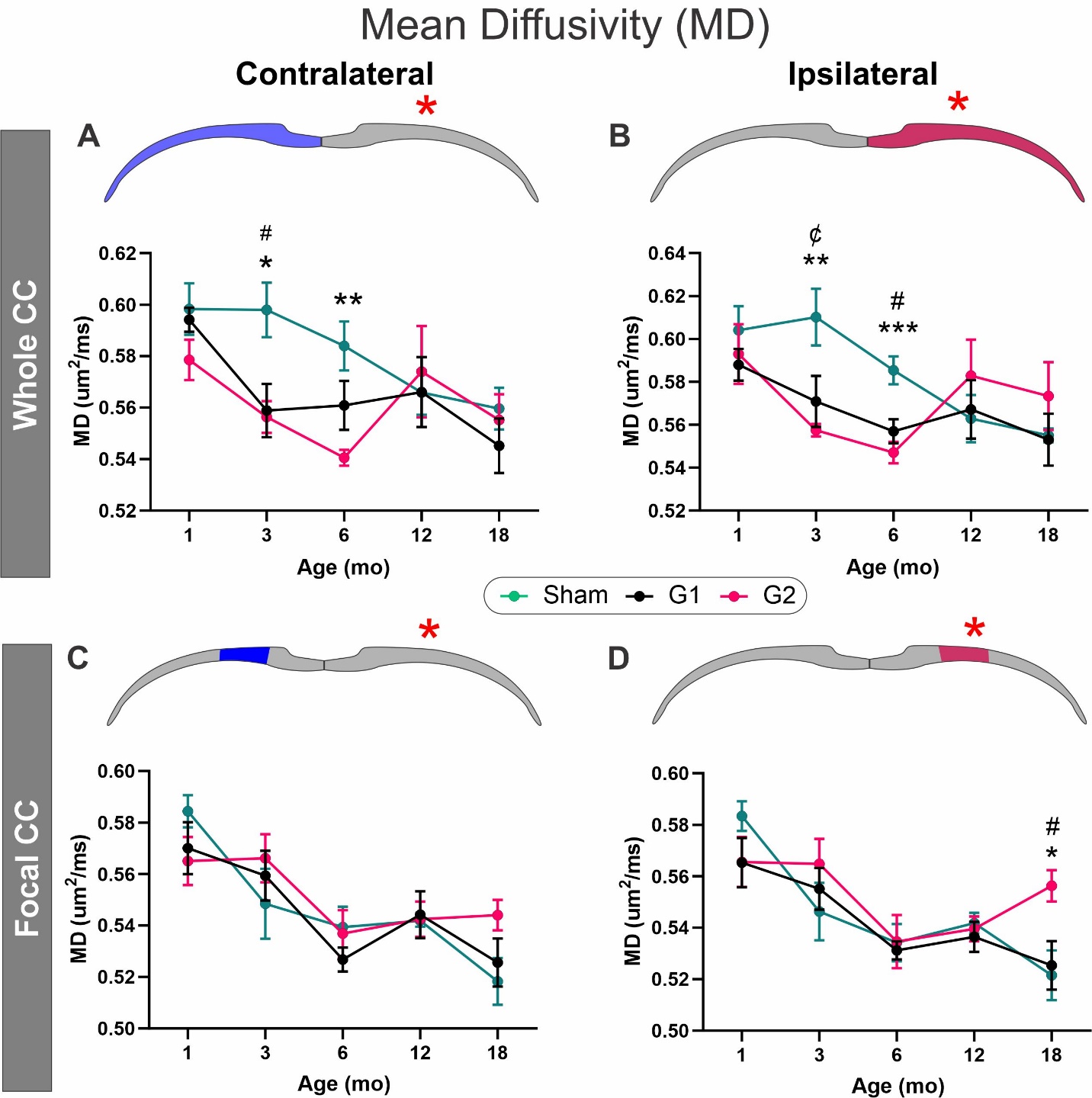


**Supplementary Figure 5. Mean diffusivity was altered in the corpus callosum in mid-life.** A) Whole CC mean diffusivity (MD) was significantly reduced in the contralateral CC with reductions at 3 and 6mpi in G2 mice compared to Shams. G1 mice were only significantly reduced at 3mpi. B) Identical to the contralateral CC there were significant differences within the whole CC with decreased MD at 3-6mpi compared to shams in both G1 and G2 mice. C) In focal CC measures no significant changes were found in the contralateral CC. D) There were also no significant ipsilateral MD changes in the focal CC measures except for G2 mice which exhibited elevated MD at 18mpi, compared to G1 and sham mice. (* p<0.05, ** p<0.01, *** p<0.001 for G2 compared to shams; # p<0.05 for G1 compared to shams; ¢ p<0.05 for comparisons between G1 and G2)


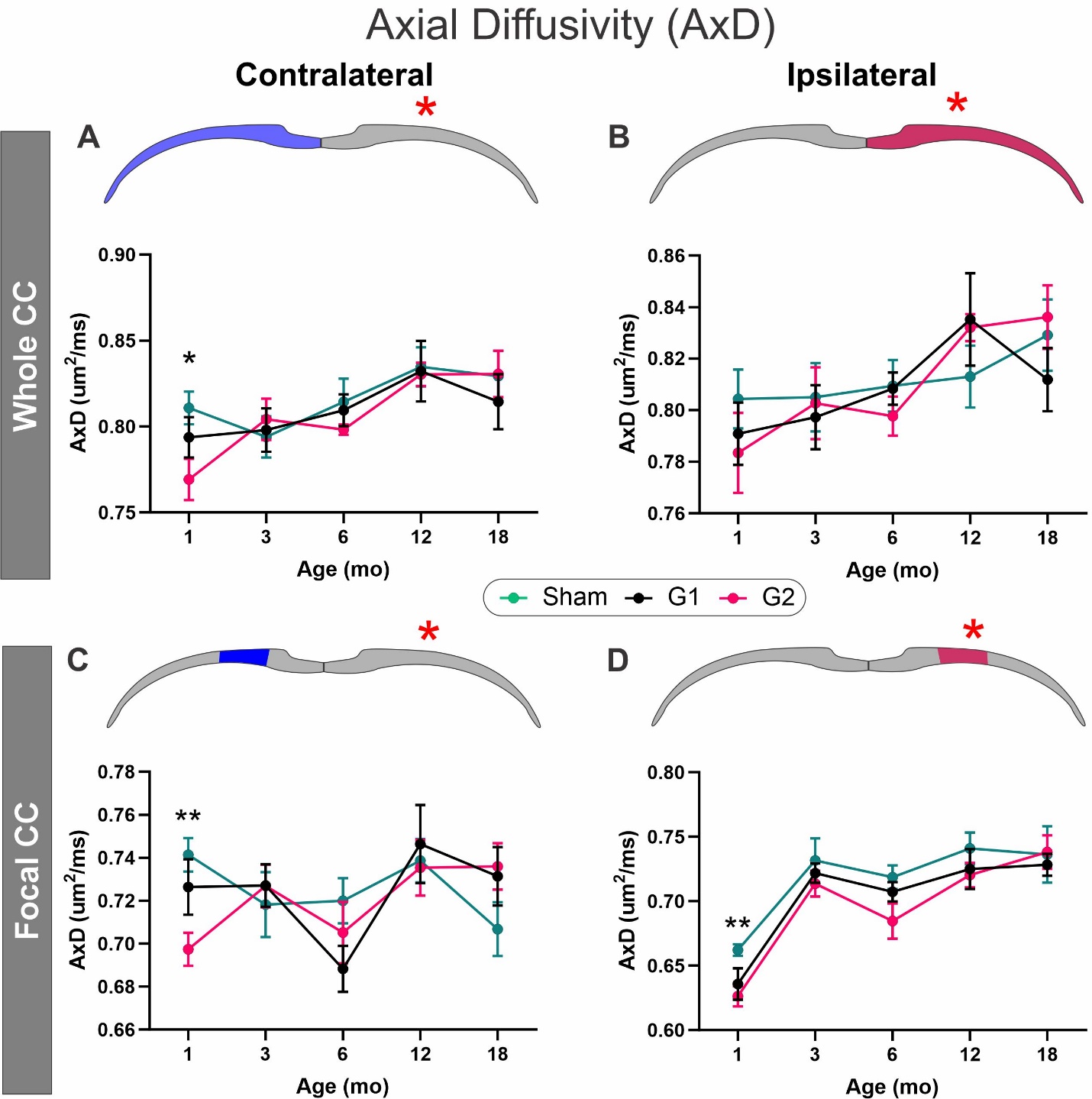


**Supplementary Figure 6. Axial diffusivity (AxD) was reduced in the corpus callosum at 1mpi.** A) Whole CC AxD was significantly reduced at 1mpi in G2 mice compared to Sham and G1 mice. B) No significant changes in AxD were observed on the ipsilateral CC at any timepoint, albeit G2 mice were reduced compared to shams (note increased variance at 1mpi). C) In focal CC measures only the G2 mice at 1mpi has significant reductions in the contralateral CC. D) Identical reductions in AxD were seen at 1mpi in the ipsilateral CC of G2 mice but not shams nor G1 mice. (* p<0.05, ** p<0.01 for G2 compared to sham)


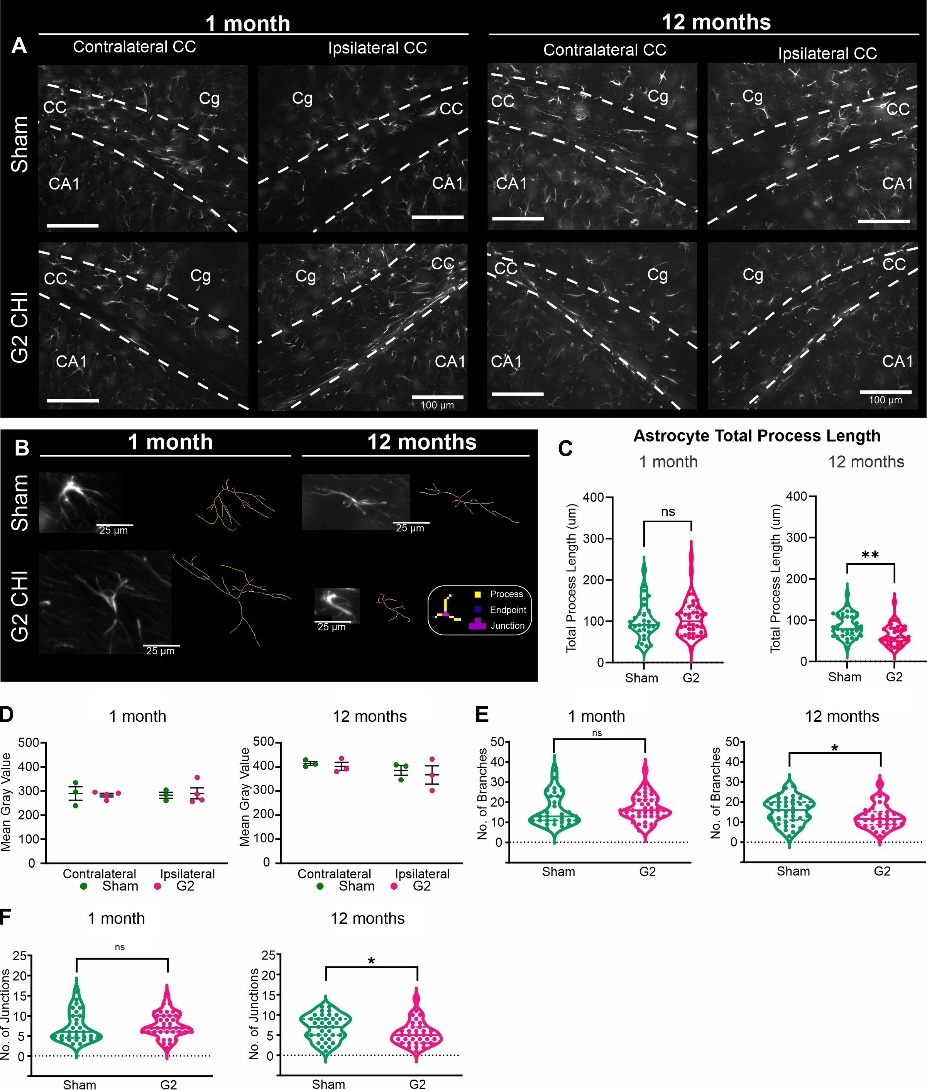


**Supplementary** **Figure 7. Astrocyte morphology is altered at 12mo after** **closed head injury (CHI) in the corpus callosum (CC).** A) Representative immunohistochemical sections stained for glial fibrillary acidic protein (GFAP) at 1 and 12mpi in shams and in Grade 2 (G2) CHI mice. (Cg – cingulum bundle, CA1 – cornu ammonis 1). B) Sample GFAP-stained astrocytes from the ipsilateral CC illustrate the change in astrocyte morphology with age (1, 12mo) and after CHI. Different morphological cell features are tagged by skeleton analysis as processes (yellow), endpoints (blue) and junctions (magenta). C) Quantitative analyses of the astrocyte total process length (μm) found no significant differences between shams and G2 CHI at 1mo after injury. However, there were significant reductions in astrocyte process length in G2 CHI in the CC at 12mpi compared to shams (** p<0.01, repeated measures Anova). Total process length was measured as the number of slab voxels. D) Mean gray value quantification showed no significant differences between sham and G2 groups at 1 and 12mpi (Kruskal-Wallis test, n=3-4 mice per group, data expressed as mean ± SEM). E) Astrocyte skeleton morphology analysis in lateral CC ROI showed a significant decrease of number of branches at 12mpi, but not at 1mpi (unpaired t-test, n=3-4 mice per group). F) Number of junctions in astrocytes was significantly reduced in G2 mice compared to sham group at 12mpi. There was no effect on the number of junctions at 1mpi (unpaired t-test, n=3-4 mice per group). * p<0.05


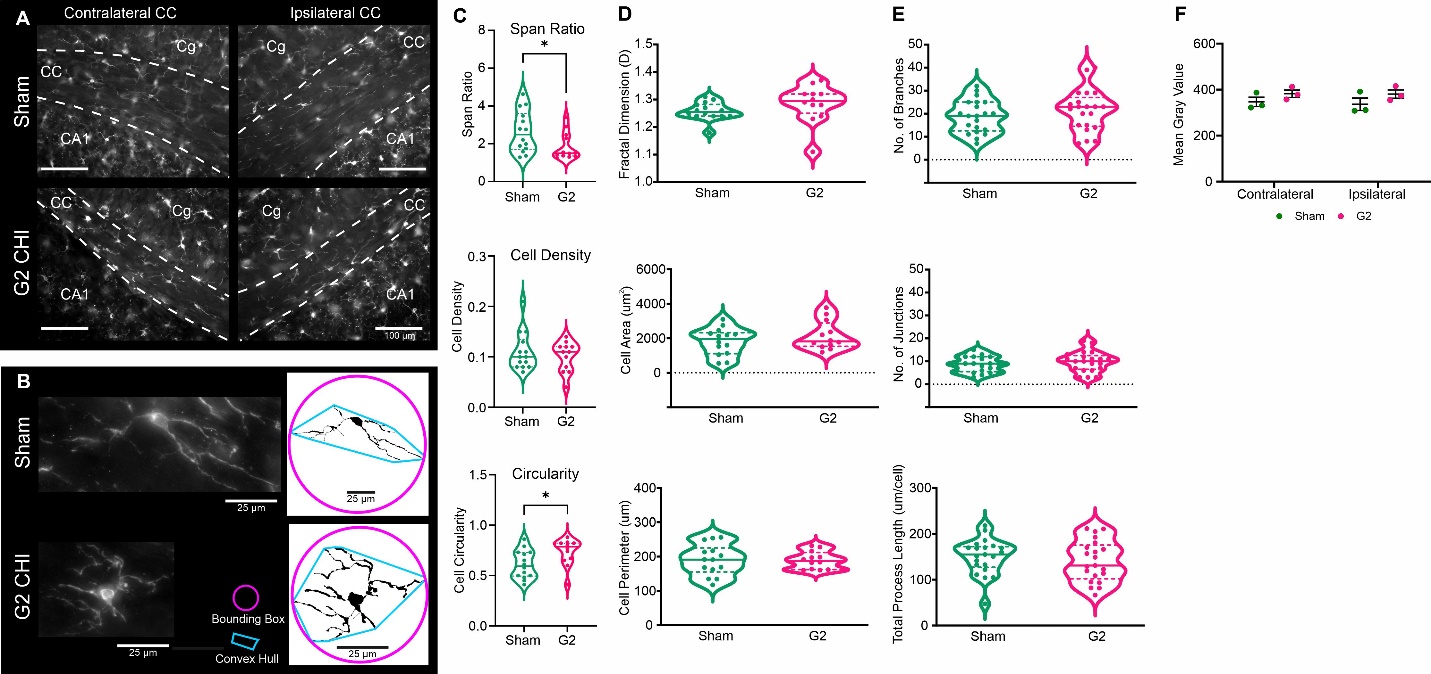


**Supplementary Figure 8. Microglial morphology in the corpus callosum (CC) is altered 12 months after Grade 2 (G2) closed head injury (CHI).** A) Representative sections from the contralateral and ipsilateral CC stained with microglial marker ionized calcium-binding adapter molecule 1 (IBA1). (Cg – cingulum bundle, CA1 – cornu Ammonis 1). B) Representative microglia from Sham and G2 CHI mice at 12mpi (left) and its schematic binary image resulting from quantitative fractal analysis (right). Convex hull (blue) and bounding circle (pink) of cells are shown. Note that the Sham microglia schematic is reduced 55%. Cells were pooled from 3 sham and 3 G2 mice. C) Microglial features such as span ratio was significantly reduced with concomitant significant increase in cell circularity of G2 CHI mice (* p<0.05). There were no significant changes in cell density between sham and G2 mice. Thus, CHI at 12mpi induces increased “roundness” of microglial cells in the CC at 12mpi compared to sham mice. D) Fractal analysis in ipsilateral CC found no significant differences between sham and G2 microglia in morphometric parameters such as fractal dimension (represents cell complexity), IBA1-positive cell area and cell perimeter (unpaired t-test, microglial cells pooled from 3-4 mice per group). E) Skeleton morphology analysis did not reveal significant differences in the number of branches, junctions and total process length (unpaired t-test, microglial cells pooled from 3-4 mice per group). F) Mean grey value quantification showed no significant difference between sham and G2 groups at 1 and 12mpi (Kruskal-Wallis test, n=3-4 mice per group, data expressed as mean ± SEM).


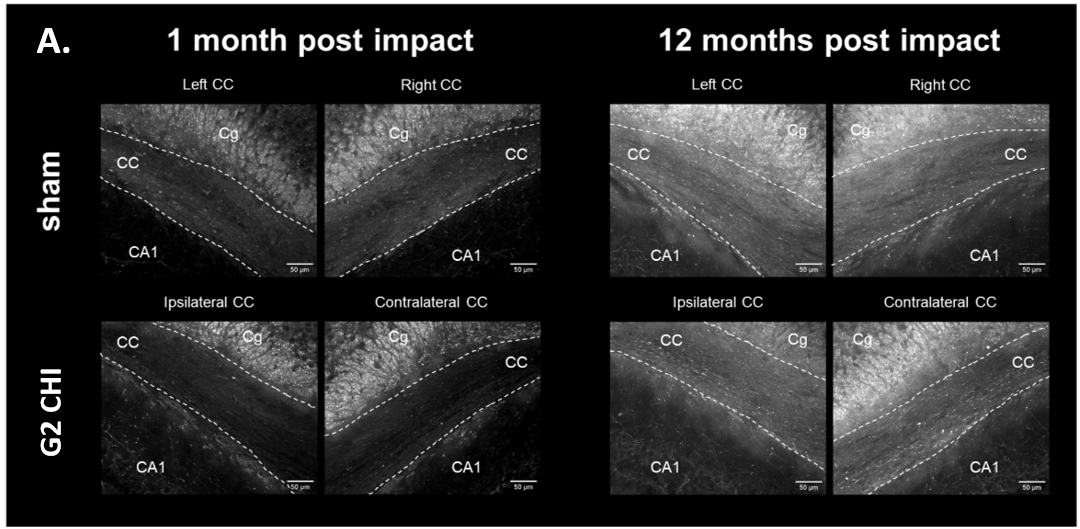

**Supplementary Figure 9. NF200 staining quantification in the corpus callosum (CC) after closed head injury (CHI).** A) Coronal brain sections from sham (top) and Grade 2 (G2) CHI mice (bottom) stained with neurofilament 200 (NF200) marker for myelinated fiber neurons at 1 (left) and 12mpi (right). B) Mean grey value quantification showed no significant difference between sham and G2 groups at 1 and 12mpi (Kruskal-Wallis test, n=3-4 mice per group, data expressed as mean ± SEM).

**References**

Ullmann, J. F., C. Watson, A. L. Janke, N. D. Kurniawan and D. C. Reutens (2013). "A segmentation protocol and MRI atlas of the C57BL/6J mouse neocortex." Neuroimage **78**: 196-203.
